# The Central-Periphery Hypothesis Revisited: Implications for Long-Term Genetic Conservation

**DOI:** 10.3390/plants14233563

**Published:** 2025-11-21

**Authors:** Rita Verbylaitė, Filippos A. Aravanopoulos, Virgilijus Baliuckas, Nikolaos Tourvas, Anna-Maria Farsakoglou, Vasiliki-Maria Kotina, Fani G. Lyrou, Aušra Juškauskaitė, Raimundas Petrokas, Vaidotas Lygis

**Affiliations:** 1 Research Centre for Agriculture and Forestry, Institute of Forestry, LT-58344 Akademija, Lithuania; virgilijus.baliuckas@lammc.lt (V.B.); ausra.juskauskaite@lammc.lt (A.J.); raimundas.petrokas@lammc.lt (R.P.); 2 Faculty of Environmental Engineering, Lietuvos Inžinerijos Kolegija Higher Education Institution, Tvirtovės al. 35, LT-50155 Kaunas, Lithuania; 3 Faculty of Agriculture, Forestry and Natural Environment, Aristotle University of Thessaloniki, P.O. Box 238, GR-541 24 Thessaloniki, Greece; tourvasn@for.auth.gr (N.T.); amfarsakoglou@gmail.com (A.-M.F.); kotinavm@for.auth.gr (V.-M.K.); lyroufani@for.auth.gr (F.G.L.); 4 State Scientific Research Institute Nature Research Centre, Akademijos Str. 2, LT-08412 Vilnius, Lithuania; vaidotas.lygis@gamtc.lt

**Keywords:** alder, spruce, genetic diversity, genetic monitoring, SSR, rear-edge populations

## Abstract

The aim of the present study was to test the central-periphery hypothesis (CPH) of evolutionary genetics in terms of genetic diversity and differentiation in *Alnus glutinosa* (L.) Gaertn., 1790 and *Picea abies* (L.) H. Karst. populations. A total of 18 nuclear SSR loci were used to evaluate genetic diversity and differentiation of two rear-edge populations of each tree species from the south-eastern edges of their distribution ranges in Greece, and two populations of each species from the core distribution area in Lithuania. Peripheral populations of *A. glutinosa* exhibited high genetic diversity (mean *Ar* = 7.99, mean *He* = 0.72) and low genetic differentiation (peripheral and core population *F_ST_* were 0.031 and 0.008, respectively). The genetic diversity values were even higher in the peripheral populations of *P. abies* (mean *Ar* = 12.27, mean *He* = 0.78), while genetic differentiation was also low (peripheral and core population *F_ST_* was 0.013 and 0.011, respectively). Genetic differentiation between the peripheral and core regions was also low (*F_ST_* = 0.038 and *G*″*_ST_* = 0.262 for *A. glutinosa* and *F_ST_* = 0.023 and *G*″*_ST_* = 0.172 for *P. abies*). Observed heterozygosity was found to be higher in peripheral populations (0.80 on average for alder and 0.84 for spruce) than in core ones (0.72 and 0.83 on average for *A. glutinosa* and *P. abies*, respectively). On the other hand, expected heterozygosity was higher in *A. glutinosa* core populations than in its peripheral ones (0.73 vs. 0.72 on average for core and peripheral alder populations), while spruce populations were less heterozygous in the core area (0.78 vs. 0.75 on average for peripheral and core spruce populations, respectively). These results indicate only partial agreement with CPH. Rear-edge populations showed higher genetic differentiation, while their lower genetic diversity was not significantly different from that of core populations. The investigated rear-edge populations of *A. glutinosa* and *P. abies* present valuable genetic reserves of European importance. They show local adaptation and present ample genetic variation, and their effective population size will likely be sufficient for adaptive evolution in the future. Their long-term conservation status should be prioritized.

## 1. Introduction

Geographically peripheral forest tree populations are of immense importance since they serve as natural laboratories for species adaptation to global climate change [1]. This is especially true for rear-edge populations, which, despite their typically small size, persist for a long time in suitable but restricted habitats. Such populations are likely to be locally adapted and are currently among the most affected by unprecedented climate change [2]. Tree species growing in the southern areas of their distribution range are expected to be more affected [3], as climate change is predicted to manifest more strongly in the southern habitats [4].

The central-periphery hypothesis (CPH) of evolutionary genetics suggests that marginal populations of living organisms should be less genetically diverse and be more differentiated than the core ones [5]. For plants, as reviewed by Eckert et al. [6], this pattern is prevailing and, on average, within-population genetic diversity declines, and among-population differentiation increases from the centre to the periphery of the species’ natural range [6]. However, some species do not follow this trend [7,8,9]. The pattern of geographic variation in population genetic diversity and differentiation is likely (but not exclusively) determined by historical factors (such as climate change events, e.g., glaciation) [6,10,11,12], and subsequent population and species migration events [13,14], and references therein [15]. Typically, clines demonstrating decreased genetic diversity with increased distance from refugia are seen when postglacial range expansions occur [16,17]. Nevertheless, some species spreading from different glacial refugia form secondary contact zones that result in an increase in genetic diversity [18].

Black alder (*Alnus glutinosa* (L.) Gaertn., 1790) is a keystone nitrogen-fixing species of riparian and water-logged habitats [19,20,21,22]. It is distributed across Europe, from Scandinavia to the Mediterranean and parts of North Africa [19]. Black alder is among the most susceptible tree species to climate change because rising temperatures and increased droughts are expected to primarily affect water-demanding and drought-sensitive species. It is well established that *A. glutinosa* harbours extensive genetic diversity [18,22,23,24,25,26,27]. The most genetically diverse *A. glutinosa* populations are found in Scandinavia and Central Europe [18], as well as in Lithuania [27]. Secondary contact zones, where admixture between genetic lineages from Eastern and Western Europe occurred, are considered to be the basis for the high genetic diversity observed in core populations of this tree species [18]. Most *A. glutinosa* populations are diploid; however, tetraploidy has also been found [23] to an estimated level of almost 20% of European populations [25]. Diploid populations prevail in Europe, North Africa, and western Asia, and tetraploid populations were found in the Iberian Peninsula, Morocco, and in the Dinaric Alps extending to the south-western Balkans, with the latter considered to be of autopolyploid nature [25].

Norway spruce (*Picea abies* (L.) H. Karst.) is one of the most important forest tree species in Europe, both economically and ecologically [28]. Norway spruce is a dominant, widespread tree species in North European boreal forests as well as in subalpine areas in the Alps and the Carpathian Mountains. Its distribution range covers the central part of Europe and spans up to the Ural Mountains [29,30]. This species, due to its shallow root system, is particularly susceptible to the heat, drought, and storms that are becoming more severe under the current climate change [31,32]. Reports on the genetic diversity of this widespread forest tree vary: Numerous studies across Europe using genetic markers [33,34,35] indicate its high genetic diversity, while Wang et al. [36], based on whole genome sequencing data, assert that *P. abies* has rather low genomic diversity, probably due to several genetic bottlenecks. Milesi et al. [11], however, place Norway spruce among the tree species with intermediate diversity. Its populations and ecotypes are usually reported to have low but significant genetic differentiation [11,33,35,37,38,39,40] caused by the postglacial recolonization, where the southern part of the species distribution led to a significant structuring, while the northern range was recolonized from a single refugium in Russia [41].

The aim of the present study was to test the central-periphery hypothesis by evaluating genetic diversity and differentiation of the south-eastern rear-edge *A. glutinosa* and *P. abies* populations located in Greece, comparing it to the genetic diversity of core populations located in Lithuania, and assessing the genetic potential of the south-eastern populations of both tree species for long-term conservation.

## 2. Results

For the *A. glutinosa* population analysis, we retained 18 loci, while two loci (Ag20 and Ag23) were excluded due to poor amplification (36 and 32 individuals have not scored in these loci, respectively). For analysis of the *P. abies* population, all 18 loci were used. Most of the analyzed *A. glutinosa* loci had no null allele presence. The highest null allele presence was found for the Ag14 locus (0.097), while other loci that contained null alleles (A22, A26, and Ag13 showed their frequency lower than 0.090. For *P. abies*, only three loci showed the presence of null alleles: paGB8 (0.091), PAAC23 (0.069), and EATC3C05 (0.161). As most loci showed no null allele presence, no relevant correction method was applied.

All loci retained for the *A. glutinosa* population analysis were polymorphic. They generated 5–24 alleles each, with an average of 13.6 alleles per locus. Out of 245 alleles in total, 114 were absent or rare (frequency ≤ 0.05), 43 were common (frequency > 0.05) in all studied populations of this tree species, and 56 were private (i.e., present in only one population). Even though four alleles were found to be rare or absent in core alder populations, but were common in peripheral ones, and thirteen alleles were found to be common in their core populations but absent or rare in the peripheral populations, no pattern of allelic distribution between core and peripheral alder populations was detected.

All loci used for the *P. abies* population analysis were polymorphic. They generated 9–39 alleles each (9–38 and 11–39 alleles for expressed sequence tags (EST) and nuclear loci, respectively), on average 22.94 alleles (16.55 and 29.33 alleles for EST and nuclear loci, respectively). Out of the detected 413 alleles, 149 were found in EST, and 264 in nuclear loci. Of all alleles found in *P. abies* populations, 275 were absent or rare (92 and 183 alleles for EST and nuclear loci, respectively), 42 were common (22 and 20 alleles for EST and nuclear loci, respectively), and 137 were private (49 and 88 alleles for EST and nuclear loci, respectively). Peripheral populations of *P. abies* had 28 common alleles that were absent (5) or rare in its core populations, and 20 common alleles were found in the core populations that were absent (3) or rare in the peripheral ones.

The core populations of *A. glutinosa* appeared to be more genetically diverse than the peripheral populations (Table 1). The estimated mean different allele number was 10.16 ± 0.47 for the core populations, while for the peripheral ones, it was lower, 8.50 ± 0.55. The effective number of alleles and allelic richness also followed the same trend—*Ae* was found to be 4.91 ± 0.35 for core populations and 4.42 ± 0.32 for peripheral populations, while *Ar* varied from 7.99 ± 0.52 in peripheral populations to 9.39 ± 0.43 in core populations. Differences between core and peripheral populations in the number of different alleles, number of effective alleles, allelic richness, and number of rare and private alleles were not statistically significant, as shown by *t*-tests (File S1). On the contrary, core populations of *P. abies* were found to show slightly less variation as compared to the peripheral ones (Table 2). The mean effective number of alleles and allelic richness were 5.34 ± 0.51 vs. 6.98 ± 0.81 and 9.74 ± 0.66 vs. 12.27 ± 0.97 for core and peripheral spruce populations, respectively. The mean number of different alleles in the peripheral *P. abies* populations was slightly higher than in its core populations (13.47 ± 1.12 vs. 12.75 ± 0.96).

For both tree species, observed heterozygosity was found to be higher in peripheral populations (on average 0.80 ± 0.03 for *A. glutinosa* and 0.84 ± 0.03 for *P. abies*) than in core ones (on average 0.72 ± 0.03 for *A. glutinosa* and 0.83 ± 0.03 for *P. abies*). On the other hand, expected heterozygosity was higher in alder core populations (Table 1), while spruce populations were less heterozygous in the core area (Table 2). The mean inbreeding coefficient *F_IS_* for *A. glutinosa* core populations was close to zero (0.03 ± 0.03), indicating random mating. The peripheral populations of this tree species showed some homozygote deficiency (*F_IS_* = −0.08 ± 0.05). The number of migrants per generation in core alder populations was very high; much higher than that in the peripheral ones (mean *Nm* = 50.54 ± 9.19 vs. 12.44 ± 2.00, respectively; Table 1). The mean inbreeding coefficient for *P. abies* populations was close to zero in peripheral populations (*F_IS_* = −0.05 ± 0.05), while its core populations showed excess heterozygosity (mean *F_IS_ =* −0.10 ± 0.05). The average number of migrants was higher for peripheral spruce populations as compared to core ones (*Nm* = 38.41 ± 8.92 vs. 32.10 ± 5.19, respectively; Table 2).

Effective population size calculated for *A. glutinosa* core populations varied from 119.7 to 180.6, while in its peripheral populations, the values were smaller: 87.6 in Mouries (GR-AG-01) and 48.8 in Lake Chimaditis (GR-AG-02) (Table 1). Norway spruce populations were found to have a higher effective population size than alder populations. Specifically, the central populations (LT-PA-IGN and LT-PA-DRU) had an effective population size of 103 and 458, respectively, while the peripheral populations presented an infinite effective population size. Despite some numerical differences, no statistical differences (at *p* < 0.05) were found between the investigated core and peripheral populations for either tree species. The only exception was a difference in the inbreeding coefficient (*F_IS_*) for *A. glutinosa* (File S1 Table S5).

**Figure 1:**
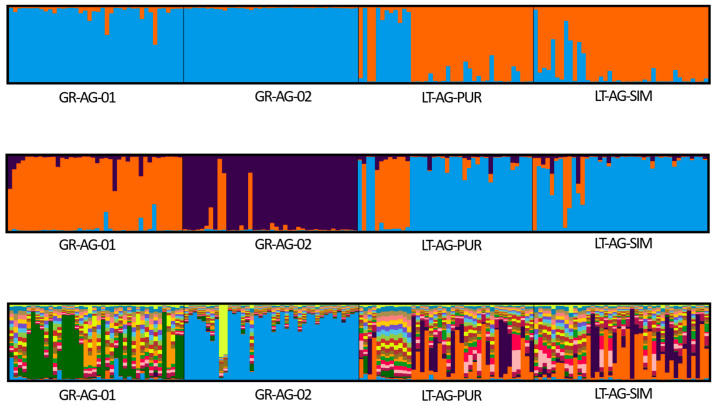
Genetic structure of *Alnus glutinosa* (L.) Gaertn., 1790 populations as identified by Bayesian analysis results. Each tree in the diagram is represented by a vertical line; the colour denotes part of the genome that belongs to each of the two clusters (K) (**top histogram**), three clusters (**middle histogram**), or 21 clusters (**bottom histogram**). Populations from Greece (GR-AG-01 and GR-AG-02; see Table 5 for more information) represent peripheral populations, and populations from Lithuania (LT-AG-PUR and LT-AG-SIM; see Table 5 for more information) represent core ones.

**Figure 2:**
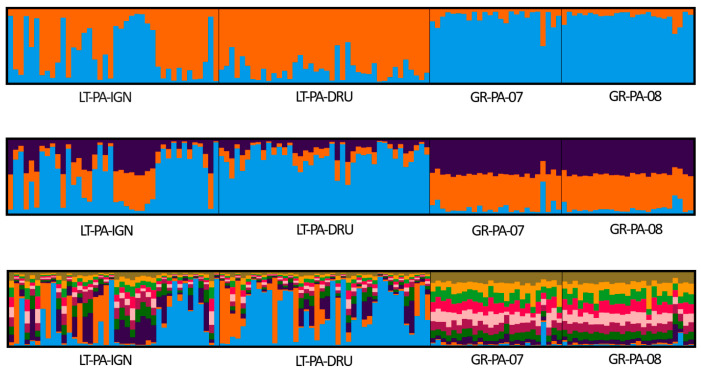
Genetic structure of *Picea abies* (L.) H. Karst. populations as identified by Bayesian analysis results. Each tree in the diagram is represented by a vertical line; the colour denotes part of the genome that belongs to each of the two clusters (K) (**top histogram**), three clusters (**middle histogram**), or ten clusters (**bottom histogram**). Populations from Lithuania (LT-PA-IGN and LT-PA-DRU; see Table 5 for more information) represent core populations, and populations from Greece (GR-PA-07 and GR-PA-08; see Table 5 for more information) represent peripheral ones.

Based on the results of the Bayesian clustering approach implemented in the structure, all analyzed *A. glutinosa* and *P. abies* populations were assigned to two genetic groups (Figure 1 for *A. glutinosa* and Figure 2 for *P. abies*). The most probable number of clusters, as determined by the Δ*K* criterion [42], was K = 2 for both species. For *A. glutinosa*, the next most probable number of clusters was K = 3 and K = 21 (see File S2 Figure S1 for likelihood probabilities). For *P. abies*, the next most probable number of clusters was K = 3 and K = 10 (see File S2 Figure S2 for likelihood probabilities).

The genetic differentiation of the investigated populations was small, though statistically significant for both tree species (Table 3 and Table 4, respectively). Higher pairwise *F_ST_* values were exhibited between peripheral *A. glutinosa* populations than between its core populations (0.031 vs. 0.008, respectively), and, logically, *F_ST_* values between core and peripheral populations were even higher (ranged between 0.037 and 0.058; Table 3). For *P. abies*, the pairwise differentiation between regional populations was of similar magnitude (*F_ST_* = 0.011 and 0.013 within core and peripheral populations, respectively), while the differentiation between core and peripheral populations was higher (ranging between 0.023 and 0.035; Table 4). Population differentiation, when using Nei’s standardized *G*″*_ST_* corrected for bias when the number of populations is small, revealed a remarkably similar trend, but, in general, of higher magnitude (Table 3 and Table 4). It should be noted that for *A. glutinosa,* the differentiation between the two peripheral populations (*G*″*_ST_* = 0.178) was of similar magnitude to the differentiation between its core and peripheral populations (*G*″*_ST_* = 0.234–0.357), while the differentiation between the two core populations was very small (*G*″*_ST_* = 0.008) and non-significant (Table 3). For *P. abies*, the *G*″*_ST_* differentiation values both within its core and within peripheral populations were of similar magnitude (*G*″*_ST_* = 0.043 and 0.030, respectively), yet considerably lower than between its core and peripheral populations (*G*″*_ST_* = 0.144–0.230) (Table 4). When individuals from the same regions were pooled together, the differentiation for *A. glutinosa* was found to be *F_ST_* = 0.038 ± 0.012 and *G*″*_ST_* = 0.262 ± 0.085 with the probability *p* = 0.001 after 1000 permutations. The differentiation between the core and peripheral regions for *P. abies* was *F_ST_* = 0.023 ± 0.005 and *G*″*_ST_* = 0.172 ± 0.049 with the probability *p* = 0.001 after 1000 permutations.

**Figure 3:**
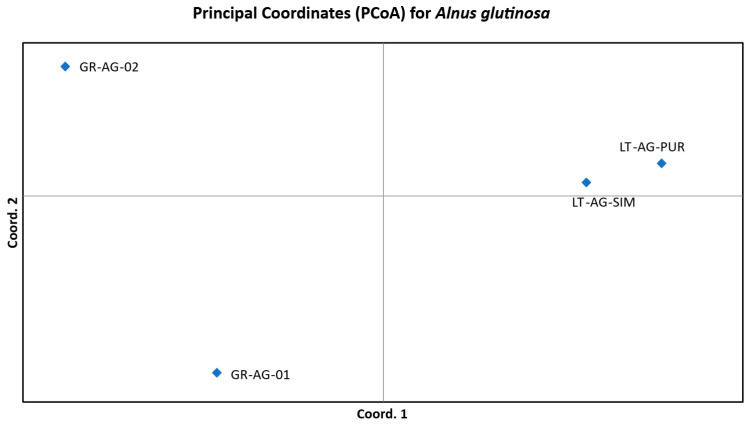
Principal coordinate analysis of the investigated core and peripheral *Alnus glutinosa* (L.) Gaertn., 1790 populations, based on SSR data. The first two coordinates explain 99.2% of the total variation. See Table 5 for the explanation of population names.

**Figure 4:**
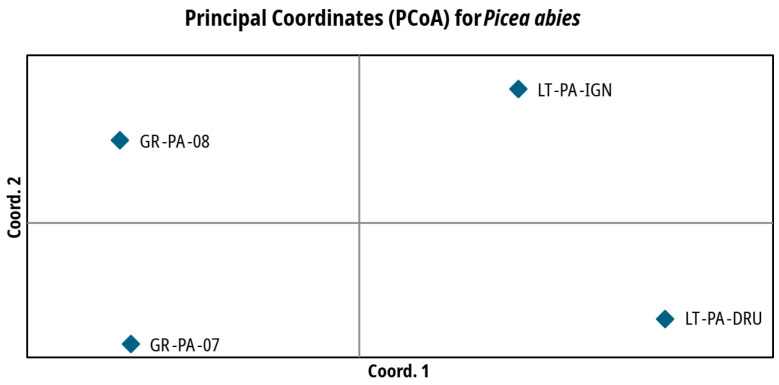
Principal coordinate analysis of the investigated core and peripheral *Picea abies* (L.) H. Karst. populations, based on SSR data. The first two coordinates explain 93.43% of the total variation. See Table 5 for the explanation of population names.

The interrelations of the investigated *A. glutinosa* and *P. abies* populations in multivariate space after the PCoA analysis are, respectively, depicted in Figure 3 and Figure 4. The PCoA further confirms Bayesian clustering data and population differentiation data (Table 3 and Table 4 for *A. glutinosa* and *P. abies*, respectively). PCoA places core *A. glutinosa* populations close to each other, while peripheral populations are placed at approximately the same distance away from each other as well as from the two core populations (Figure 3). PCoA of *P. abies* populations places them much closer to each other in the coordinate field and distributes all four investigated populations almost equally (Figure 4).

## 3. Discussion

The herein-investigated peripheral populations of *A. glutinosa* and *P. abies* from Greece represent the most south-eastern rear-edge populations of these tree species in Europe. These populations, growing in Natura 2000 sites, are genetically diverse and exhibit high levels of heterozygosity. When compared to core populations from Lithuania, the Greek population presents a higher number of private alleles (the exception being GR-AG-02), while the number of rare alleles is similar across all populations within a species. The majority of mean genetic diversity measures (*Ae*, *Ar*, and *He*) estimated for *A. glutinosa* were lower in its peripheral populations, while *Ho* was found to be lower in the core populations (Table 1). In *P. abies*, the situation was the opposite—mean genetic diversity measures (*Ae*, *Ar*, *Ho*, and *He*) were higher in peripheral populations compared to the core ones (Table 2). However, the core and peripheral populations only exhibited numerically higher values of genetic diversity parameters, yet the differences were not statistically significant (File S1).

Genetic diversity indices of the peripheral *A. glutinosa* populations found in the present study are somewhat higher than those found in other population studies of this tree species. For example, in the study by Havrdova et al. [18], the only Greek population studied with SSRs, showed a lower value in *Ar* and the same *He* value as the present study. In the Belgium–Luxembourg–France cross-border area, the average allelic richness was found to be *Ar* = 6.23, while observed and expected heterozygosity were found to be *He* = 0.62 and *He* = 0.64, respectively [26]. In Northern Ireland *A. glutinosa* populations, *Ar* varied from 3.79 to 5.06, *Ho* from 0.53 to 0.68, and *He* from 0.58 to 0.71 [24]. High genetic diversity found in rear-edge *A. glutinosa* populations from Greece is not surprising, as one of this species’ refugia during the last ice age was reported to be in the Balkan peninsula [18]. Some homozygote deficiency (*F_IS_* = −0.15 ± 0.07) was found in the Greek population of Lake Chimaditis (GR-AG-02). The negative value of this coefficient might be associated with higher selection pressure at the edge of the species distribution range [43]. The results of studies including *A. glutinosa* core populations indicate the absence of inbreeding: *F_IS_* = 0.04 was reported in the European continental mainland by Mingeot et al. [26], *F_IS_* = 0.077 in Northern Ireland by Beatty et al. [24], and *F_IS_* = 0.03 ± 0.03 in Lithuania (the present study; Table 1).

The analysis of *A. glutinosa* peripheral populations showed no presence of triploid or tetraploid individuals. As the presence of mixed-ploidy individuals is a rare event [25], we conclude that Mouries (GR-AG-01) and Lake Chimaditis (GR-AG-02) populations are diploid. In their extensive study of European *A. glutinosa* populations, Mandák et al. [25] found that its diploid and tetraploid populations are almost parapatric, and argued that the distribution differences between the populations with different ploidy levels are due to different ecological tolerance, with tetraploids being more tolerant to a wider range of ecological conditions than diploids. The peripheral *A. glutinosa* populations studied by us occupy typical habitats of this tree species in central-northern Greece, and therefore, it is suggested that extension of the tetraploid *A. glutinosa* populations from the Dinaric Alps to south-western Greece, as proposed by Mandák et al. [25], did not yet cover the territories investigated in the present study.

Genetic diversity indices found by us for *P. abies* populations are in accordance with those published in previous studies, where *He* was found to vary from 0.62 in a Serbian study [35] to 0.92 in Slovenia [34], with other studies reporting *He* values in between [33,37,38,39,40,44].

The genetic differentiation within the investigated core and peripheral *A. glutinosa* populations was generally low, with a certain variation among populations (Table 3). The two core populations of this tree species showed extraordinarily little differentiation (*F_ST_* = 0.008, and *G*″*_ST_* = 0.008), which was about 4–22 times less than the differentiation found between the two peripheral populations (*F_ST_* = 0.031 and *G*″*_ST_* = 0.178). Despite this major disparity in the two differentiation values, rendered by the differential influence of the high SSR mutation rate on the latter [45], both showed several-fold greater differences in the peripheral populations compared to the core ones. We consider that the population differences cannot be attributed to a recent or past disturbance: all herein-investigated *A. glutinosa* populations have been under protection already for decades (see Table 5), and we have not detected any recent bottlenecks by employing the Bottleneck (vs. 1.2.02) software [46]. The finding of much higher population differentiation in the peripheral *A. glutinosa* populations supports the CPH (sensu Mayr [5]).

The generally low differentiation observed between the pooled Greek and Lithuanian *A. glutinosa* populations (average *F_ST_* = 0.038, *G*″*_ST_* = 0.262) was likely due to common ancestry. Based on the results of their comprehensive study on the postglacial history of European *A. glutinosa* populations, Havrdová et al. [18] suggested that current Northeastern European populations (including Lithuania) have been formed as a result of postglacial migration from the eastern part of the Balkan Peninsula through the Carpathians to the Baltic and Northeastern European plains with a high likelihood of linear admixture from the refugium located in Belarus and western Russia [25]. In the present study, the low differentiation between the peripheral and core populations was confirmed by the Bayesian clustering approach, where the most probable number of clusters for *A. glutinosa* was found to be two (File S2 Figure S1). The peripheral populations were assigned to the first cluster, while individuals from core populations were assigned with different probabilities to the first or second genetic clusters. These results, based on a model-based Bayesian approach, are concordant with the exploratory multivariate distance-based approach of PCoA. In a high goodness-of-fit PCoA (99.2% of the total variation explained in the first two axes), the Greek populations are well separated from the core populations forming two distinct clusters (Figure 3).

For *P. abies*, the observed genetic differentiation was small both between core and peripheral populations, which is in agreement with the results of other studies [11,33,35,37,38,39,40]. The differentiation between peripheral spruce populations from Greece is even smaller than from Lithuania based on *G*″*_ST_* (Table 4). The peripheral spruce populations investigated in this study are geographically close to each other but are from different elevations. The proximity of the sampling sites in Greece (approx. 7 km) might have caused the small differentiation between the two populations. Populations from Lithuania are approx. 200 km apart; therefore, the slightly higher differentiation between the populations is not surprising. Similarly to *A. glutinosa*, the genetic differentiation between the pooled peripheral and core *P. abies* populations was low (the average *F_ST_* = 0.023 and *G*″*_ST_* = 0.172). Milesi et al. [11], after analyzing 26 populations from 13 European countries, have found four major genetic clusters. Genome capture data revealed that *P. abies* populations from Greece are admixed with those of northern Europe and Scandinavia. Moreover, Milesi et al. [11], in their comparative population genomic analyses for seven widely distributed and ecologically contrasting European forest tree species (that included *P. abies*), indicated that the geographic distribution of genetic diversity followed the south–north latitudinal gradient and did not fit CPH. Our findings were further supported by the Bayesian clustering approach, where the most likely number of clusters was found to be two (File S2 Figure S2). However, the clustering, as well as PCoA results (Figure 4), show clear differences between the core and peripheral *P. abies* populations.

The results of the present study for *P. abies* clearly do not follow the general pattern for CPH. Genetic diversity parameters found for populations of this tree species were higher in peripheral populations, while population differentiation was higher in the core area of its distribution. The differences in genetic diversity parameters between the core and peripheral populations may be due to different postglacial refugia and subsequent colonization routes, where Lithuanian populations belong to the northern part of the *P. abies* distribution, while Greek populations most likely survived the last glacial maximum somewhere in the Balkan mountains [41].

In the present study, the rear-edge populations of *A. glutinosa* showed higher genetic differentiation, but lower genetic diversity (yet the difference was not statistically significant) compared to its core populations. While in peripheral *P. abies* populations, the genetic diversity was found to be higher than in the core ones, and the difference was also non-significant. Comparable results were reported for rear-edge wild cherry (*Prunus avium* L.) populations from northern Greece [47]. The lack of significance in genetic diversity differences between central and peripheral populations was also noted by Eckert et al. [6].

## 4. Materials and Methods

### 4.1. Study Sites and Sampling

**Figure 5:**
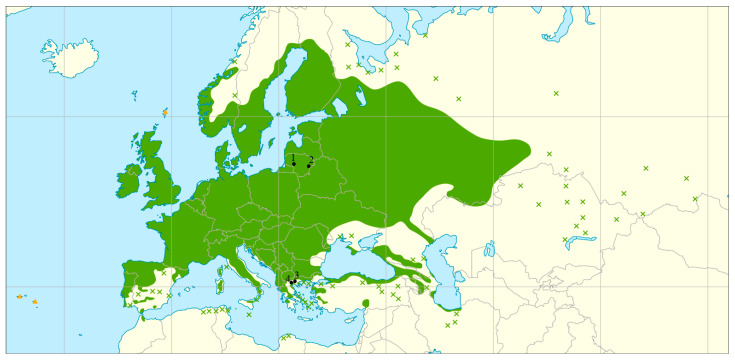
A map showing natural distribution area of *Alnus glutinosa* (L.) Gaertn., 1790 (compiled by members of the EUFORGEN network [52]) and four sampled locations. Sampled Lithuanian populations (black dots 1 and 2) represent core populations (1—LT-AG-SIM, 2—LT-AG-PUR) and sampled Greek populations (dots 3 and 4)—peripheral ones (3—GR-AG-01, 4—GR-AG-02).

**Figure 6:**
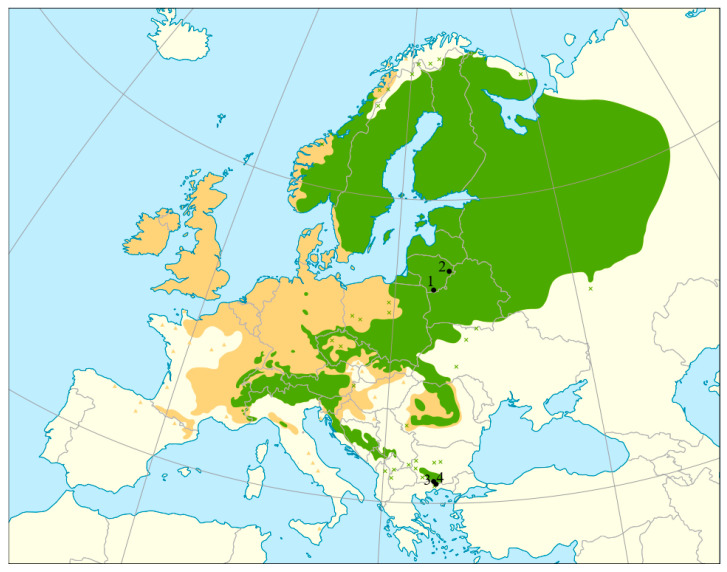
A map showing natural distribution area of *Picea abies* (L.) H. Karst. (compiled by members of the EUFORGEN network [53]) and four sampling locations. Sampled Lithuanian populations (black dots 1 and 2) represent core populations (1—LT-PA-DRU, 2—LT-PA-IGN) and sampled Greek populations (dots 3 and 4)—peripheral ones (3—GR-PA-07, 4—GR-PA-08).

In the present study, we investigated four *A. glutinosa* and four *P. abies* populations. For both species, two peripheral populations from Greece and two from the core distribution area in Lithuania were investigated (Table 5; Figure 5 for *A. glutinosa* and Figure 6 for *P. abies*). The peripheral populations comply with all categories of marginality indices as presented by Picard et al. [48]: environmental marginality (strong climatic differences between core and peripheral areas), peripherality (populations at the rear edge of the species’ natural distribution), and historical marginality (proximity to rear edge along the latitudinal range), while the core populations, in contrast, do not comply with any of the marginality indices of Picard et al. [48]. The peripheral populations of *A. glutinosa* belong to the Balkan refugium [23,25], while *P. abies* populations are from the Elatia area of the Southern Rodopi mountains refugium and are in fact the most south-eastern populations of the species’ natural distribution [35,41,49]. The core populations selected for this study represent different provenance regions for *A. glutinosa* and *P. abies* in Lithuania. Lithuanian *A. glutinosa* populations may be regarded as typical populations of this tree species in the region because their average genetic diversity values are similar to those of eight other previously studied populations from this region [27].

All populations included in the present study were located in protected areas—either in genetic reserves or Natura 2000 territories (Table 5). All sampled populations complied with minimum size requirements for dynamic in situ genetic conservation units (GCUs) of forest tree genetic diversity. The Greek populations complied with Case 2 population size (marginal populations that harbour at least 50 reproducing trees), while Lithuanian populations complied with Case 1 (500 or more reproducing trees) [50,51].

In the present study, a total of 160 *A. glutinosa* tree individuals were analyzed, of which 80 originated from peripheral and 80 from the core populations of this tree species. As regards *P. abies*, 50 and 80 tree individuals from peripheral and core populations were sampled, respectively (Table 5). In each population, 40 trees were sampled except for *P. abies* in Greece, where only 25 mature trees were sampled. At each sampling site, 25–32 of the sampled trees were mature (of reproducing age), while the rest were of juvenile age (2–15 years old), all originating from the natural regeneration. Details on sample size and population data are given in Table 5. In core populations, samples from trunks of the mature trees were taken with an electric drill, making a 2–3 cm-deep borehole and collecting the resulting sawdust in 2 mL sterile sample tubes. From each sampled juvenile tree, three to four *A. glutinosa* leaves or a 5–10 cm-long *P. abies* twig with needles were collected and placed separately in paper sampling bags. Samples collected in the core populations were transported to the laboratory on the same day of sampling and placed in the freezer (−20 °C) until DNA extraction. In peripheral populations, leaf samples (three to four leaves per tree) from mature and juvenile trees of *A. glutinosa*, and 5–10 cm-long *P. abies* twigs with needles were collected and placed separately in paper bags, each containing 5 g of desiccating silica gel. The silica gel was replaced, if necessary, until the sampled material was totally dry. The sampled material was kept in a cool, dry place until DNA extraction.

The DNA extraction from *A. glutinosa* and its simple sequence repeat (SSR) analysis was carried out as described in Verbylaitė et al. [27]. For DNA extraction from the desiccated material (peripheral populations), 20 mg of dry leaves or needles from each sampled tree was used. For analysis of *A. glutinosa*, DNA samples were used from 20 nuclear SSR loci: A2 [54], A22 and A26 [55], A10 [56], A35 and A38 [57], A7 [58], A37 [59], Ag30, Ag05, Ag10, Ag14, Ag27, Ag35, Ag13, Ag25, Ag09, Ag20, Ag01, and Ag23 [60]. Polymerase chain reaction (PCR) was performed in two multiplex reactions, as described in Drašnarová et al. [61] and Lepais and Balces [60] (additional primer and loci information is presented in File S3). For analysis of *P. abies* DNA samples, 18 polymorphic SSR loci were employed (paGB3, paGB8, pgGB5, pgGB7, and prGB1 [62], PA0034 and PA0043 [63], WS0015.I04 and WS0019.F22 [64], SpAC1H8 [65], UAPgAG150A, UAPgCT3, and UAPgCA24 [66], EAC6C02 and EAC7H07 [67], EATC3C05 [68], PAAC23 [69], and PGL15 [70]). Nine SSR loci, used in the present study, represent expressed regions of DNA, EST-SSR (paGB3, pgGB5, pgGB7, prGB1, PA0034, PA0043, WS0015.I04, WS0019.F22, and PAAC23). All SSR loci selected for *P. abies* analysis, except for two (prGB1 and EATC3C05), consisted of dinucleotide repeats, as those were proven to be more variable than trinucleotides [71]. Amplification of *P. abies* DNA was performed in five multiplex reactions, and an additional four loci in single-primer reactions (File S3). The PCR consisted of 40 ng genomic DNA (2 μL), 7.5 μL Kapa2G Fast Multiplex reaction mix (Kapa biosystems), 5 pmol of each fluorescently labelled forward and reverse primers, and ddH_2_O to a final volume of 15 μL. PCR conditions: 5 min initial denaturation at 94 °C; 35 cycles of 30 s at 94 °C followed by 30 s at the annealing temperature of used PCR primers (File S3), and 30 s at 72 °C; and final synthesis for 5 min at 72 °C. After amplification, the specificity of the PCR and amplification success was confirmed by running 5 μL of PCR product on a 1.5% TAE agarose gel. Fragment size was evaluated using MassRuller mix DNA standard (Thermo Fisher Scientific, Waltham, MA, USA). PCR products from single-primer amplification and multiplex A were combined in mix K, while PCR products from multiplexes B and C and multiplexes D and E were combined into mixes L and M, respectively. For each mix, an equimolar concentration of each PCR product was taken. DNA fragment size analysis for *P. abies* was performed in HCMR (Herclion Greece), while fragment size analysis for *A. glutinosa* was conducted by the company Genoscreen Innovative Genomics (Lille, France). In both cases, an ABI 3730XL DNA analyzer (Applied Biosystems, San Francisco, CA, USA) was used. Fragment size evaluation and scoring were performed using Geneious Prime computer software (Geneious Prime 2021.2 (https://www.geneious.com)).

### 4.2. Data Analysis

Scoring errors and null allele presence were checked with the Microchecker software (v2.2.3) [72]. Genetic diversity parameters for core and peripheral populations (number of different alleles (*Na*); number of effective alleles (*Ae*); allelic richness (*Ar*); observed heterozygosity (*Ho*); expected heterozygosity (*He*)) were calculated with GenAlEx (v6.51b2) software [73,74,75]. Principal coordinate analysis (PCoA) was used to visualize the genetic structure and relationships in a two-dimensional multivariate space using the GenAlEx software. Corrected for equal sample size, allele number—allelic richness and inbreeding coefficient (*F_IS_*) were calculated using the FSTAT software (v294) [76]. To calculate the effective population size (*Ne*) of each population, a point estimation method of linkage disequilibrium [77] using all frequency alleles and a parametric assessment of 95% confidence intervals (CI) was implemented using the NeEstimator software (v2.1) [78]. Significant differences in genetic parameters between core and peripheral populations were calculated using the TTEST procedure (Welsh *t*-test) for pairwise comparisons of the target groups of SAS software (Version 9.4, Cary, NC, USA, 2012). GenAlEx was also used to calculate population differentiation, *F_ST_*, and *G*″*_ST_*. Hedrick’s standardized *G_ST_* was further corrected for bias as the number of populations *k* is small [79]. Analysis of molecular variance (AMOVA) between regions, among populations, and individuals was performed by employing GenAlEx as well. Genetic differences among populations were investigated by a Bayesian approach using the software Structure v. 2.3.4 [80,81,82,83], which employs a model-based clustering algorithm. To estimate the optimal number of clusters, the delta *K* (Δ*K*) criterion [42] was used (Structure Selector computer software, https://lmme.ac.cn/StructureSelector/, accessed on 10 September 2025) [84]). The default model parameters used population priors; *K* (the number of populations tested) varied from 1 to 25, with 100 replications. For each run, 100,000 burn-in iterations and 100,000 data collection iterations were used.

## 5. Conclusions

Our results provided only partial agreement with CPH. The theoretical expectations of the CPH appear to be challenged when peripheral tree populations originate from glacial refugial areas. For *P. abies*, the results deviate from the classical CPH pattern, indicating that evolutionary and demographic processes shaping genetic variation in this species may not follow a simple geographic gradient. Peripheral populations exhibited higher genetic diversity, whereas population differentiation was greater within the core range. These patterns likely reflect the influence of multiple postglacial refugia and complex recolonization dynamics, with Lithuanian populations representing the northern expansion front and Greek populations originating from long-term refugial areas in the Balkan Mountains.

In *A. glutinosa*, rear-edge populations showed higher genetic differentiation, but slightly lower genetic diversity compared to core populations, although the latter difference was not statistically significant. The observed genetic patterns in both species highlight the role of historical biogeography and local demographic stability in maintaining or reshaping genetic variation across species’ ranges, rather than a uniform decline in diversity toward range margins.

The investigated rear-edge *A. glutinosa* and *P. abies* populations represent valuable genetic resources of European importance, and their conservation status should be prioritized. These populations exhibit signs of local adaptation and maintain substantial genetic variation, suggesting that their effective population size will likely be sufficient to support adaptive evolution under future environmental change. To ensure their long-term preservation, dynamic conservation measures—such as the establishment of GCUs and the implementation of continuous genetic monitoring [27,85]—should be applied to *A. glutinosa* populations from Mouries (GR-AG-01) and Lake Chimaditis (GR-AG-02), as well as *P. abies* populations from the Elatia area of the Southern Rodopi mountains.

Moreover, there is an urgent need to extend the EUFORGEN *A. glutinosa* conservation network southwards, as no GCUs currently exist for this species in Southern Europe [52]. While the Mouries population is well protected in practice, the Lake Chimaditis population requires active management intervention, as grazing is currently impeding natural regeneration and poses a direct threat to its long-term viability. Specific measures should, therefore, be implemented to promote regeneration and population stability.

The expansion of the *P. abies* conservation network to include Greece is also of high importance. At present, no GCUs have been designated for this species in the country, despite the presence of the most south-eastern marginal and potentially unique populations there [53].

## Figures and Tables

**Table 1:** Genetic diversity parameters with indicated standard errors for the studied *Alnus glutinosa* (L.) Gaertn., 1790 populations. *Na*: number of different alleles; *Ae*: number of effective alleles; *Ar*: allelic richness, calculated on 29 diploid individuals; *Ho*: observed heterozygosity; *He*: expected heterozygosity; *F_IS_*: inbreeding coefficient; *Nm*: number of migrants per generation, calculated based on *F_ST_* values. Average *Nm* for peripheral and core populations is based on peripheral or core *F_ST_* values, respectively. *Ne:* effective population size; CI: confidence interval.

	Population						
	GR-AG-01	GR-AG-02	Mean, Peripheral	LT-AG-PUR	LT-AG-SIM	Mean, Core	Grand Mean
*Na*	9.78 ± 0.85	7.22 ± 0.60	8.50 ± 0.55	9.83 ± 0.74	10.50 ± 0.63	10.16 ± 0.47	9.33 ± 0.38
*Ae*	4.92 ± 0.53	3.92 ± 0.35	4.42 ± 0.32	4.71 ± 0.48	5.12 ± 0.54	4.91 ± 0.35	4.67 ± 0.24
*Ar*	9.15 ± 0.79	6.83 ± 0.55	7.99 ± 0.52	9.08 ± 0.64	9.70 ± 0.57	9.39 ± 0.43	9.59 ± 0.59
No. of rare alleles	85	50	117 *	84	85	118 *	149 **
No. of private alleles	24	10	37 *	11	11	45 *	–
*Ho*	0.78 ± 0.05	0.82 ± 0.04	0.80 ± 0.03	0.71 ± 0.04	0.73 ± 0.04	0.72 ± 0.03	0.76 ± 0.02
*He*	0.73 ± 0.05	0.71 ± 0.03	0.72 ± 0.03	0.72 ± 0.04	0.75 ± 0.04	0.73 ± 0.03	0.72 ± 0.02
*F_IS_*	−0.06 ± 0.04	−0.15 ± 0.07	−0.08 ± 0.05	0.03 ± 0.04	0.04 ± 0.03	0.03 ± 0.03	−0.04 ± 0.04
*Nm*	3.86 ± 0.42	3.24 ± 0.61	12.44 ± 2.00	44.16 ± 33.94	44.66 ± 33.74	50.54 ± 9.19	6.14 ± 0.77
*Ne* (CI)	87.6 (70.0–115.1)	48.8 (40.3–60.6)	67.5 (61.7–74.2)	180.6 (127.2–301.2)	119.7 (94.3–161.3)	162.2 (139.3–192.6)	–

* The number given is an actual rare allele number for the region, not a mean for peripheral/core populations. ** The number given is an actual private allele number for the total sample size, not a mean for peripheral/core populations.

**Table 2:** Genetic diversity parameters with indicated standard errors for the studied *Picea abies* (L.) H. Karst. populations. *Na:* number of different alleles; *Ae:* number of effective alleles; *Ar:* allelic richness, calculated on 20 diploid individuals; *Ho:* observed heterozygosity; *He:* expected heterozygosity; *F_IS_*: inbreeding coefficient; *Nm:* number of migrants per generation, calculated based on *F_ST_* values. Average *Nm* for peripheral and core populations is based on peripheral or core *F_ST_* values, respectively. *Ne*: effective population size; CI: confidence interval.

	Population						
	GR-PA-07	GR-PA-08	Mean, Peripheral	LT-PA-IGN	LT-PA-DRU	Mean, Core	Grand Mean
*Na*	13.33 ± 1.61	13.61 ± 1.60	13.47 ± 1.12	13.56 ± 1.25	11.94 ± 1.46	12.75 ± 0.96	13.11 ± 0.73
*Ae*	7.14 ± 1.25	6.84 ± 1.07	6.98 ± 0.81	5.69 ± 0.65	5.00 ± 0.80	5.34 ± 0.51	6.17 ± 0.49
*Ar*	12.22 ± 1.37	12.32 ± 1.37	12.27 ± 0.97	10.44 ± 0.84	9.03 ± 0.98	9.74 ± 0.66	11.57 ± 1.12
No. of rare alleles	151	162	249 *	167	142	223 *	330 **
No. of private alleles	44	43	117 *	29	21	83 *	–
*Ho*	0.84 ± 0.04	0.85 ± 0.04	0.84 ± 0.03	0.83 ± 0.04	0.82 ± 0.05	0.83 ± 0.03	0.83 ± 0.02
*He*	0.78 ± 0.03	0.78 ± 0.04	0.78 ± 0.02	0.78 ± 0.03	0.73 ± 0.04	0.75 ± 0.02	0.77 ± 0.02
*F_IS_*	–0.05 ± 0.05	–0.07 ± 0.05	–0.05 ± 0.05	–0.05 ± 0.04	–0.12 ± 0.07	–0.10 ± 0.05	–0.10 ± 0.05
*Nm*	17.48 ± 9.22	17.35 ± 9.27	38.41 ± 8.92	13.32 ± 4.43	10.98 ± 5.38	32.10 ± 5.19	10.24 ± 1.42
*Ne* (CI)	∞ (805.5–∞)	∞ (147,375–∞)	50,069 (1100.8–∞)	103.0 (87.5–124.3)	458.2 (253.2–2076.7)	163.8 (147.3–183.9)	–

* The number given is an actual rare allele number for the region, not a mean for peripheral/core populations. ** The number given is an actual private allele number for the total sample size, not a mean for peripheral/core populations.

**Table 3:** Results of pairwise *Alnus glutinosa* (L.) Gaertn., 1790 population differentiation *F_ST_* (below the diagonal) and *G*″*_ST_* (above the diagonal) analyses. Probability, *p* (random > = data), based on 1000 permutations, is shown in brackets. See Table 5 for the explanation of population names.

Population	LT-AG-PUR	LT-AG-SIM	GR-AG-01	GR-AG-02
LT-AG-PUR	0.000	0.008 (0.165)	0.286 (0.001)	0.357 (0.001)
LT-AG-SIM	0.008 (0.165)	0.000	0.234 (0.001)	0.307 (0.001)
GR-AG-01	0.046 (0.001)	0.037 (0.001)	0.000	0.178 (0.001)
GR-AG-02	0.058 (0.001)	0.049 (0.001)	0.031 (0.001)	0.000

**Table 4:** Results of pairwise *Picea abies* (L.) H. Karst. population differentiation *F_ST_* (below the diagonal) and *G*″*_ST_* (above the diagonal) analyses. Probability, *p* (random > = data), based on 1000 permutations, is shown in brackets. See Table 5 for the explanation of population names.

Population	LT-PA-IGN	LT-PA-DRU	GR-PA-07	GR-PA-08
LT-PA-IGN	0.000	0.043 (0.001)	0.144 (0.001)	0.149 (0.001)
LT-PA-DRU	0.011 (0.001)	0.000	0.221 (0.001)	0.230 (0.001)
GR-PA-07	0.023 (0.001)	0.034 (0.001)	0.000	0.030 (0.020)
GR-PA-08	0.024 (0.001)	0.035 (0.001)	0.013 (0.020)	0.000

**Table 5:** Information on the investigated black alder (*Alnus glutinosa* (L.) Gaertn., 1790) and Norway spruce (*Picea abies*) populations. LT = Lithuania; GR = Greece; AG = *A. glutinosa*; PA = *P. abies*.

Sampling Locations and Population Codes	Type of Protected Area	No. of Trees Sampled; Total (Mature and Juvenile)	Population Type	Location Coordinates	
				Latitude	Longitude
Šimkaičiai, LT-AG-SIM	Genetic reserve	40 (28, 12)	Core	55.19456	22.83602
Purviniškės, LT-AG-PUR	Seed stand	40 (28, 12)	Core	55.01534	25.62753
Mouries, GR-AG-01	Natura 2000	40 (27, 13)	Peripheral	41.24923	22.77066
Lake Chimaditis, GR-AG-02	Natura 2000	40 (32, 8)	Peripheral	40.60419	21.54777
Druskininkai, LT-PA-DRU	Genetic reserve	40 (28, 12)	Core	53.96849	24.33219
Ignalina, LT-PA-IGN	Genetic reserve	40 (28, 12)	Core	55.36613	26.19626
Elatia, GR-PA-08	Natura 2000	25 (25, 0)	Peripheral	41.4850	24.3336
Tsakalos, GR-PA-07	Natura 2000	25 (25, 0)	Peripheral	41.5302	24.2835

## Data Availability

The SSR genotype data matrix is available from the corresponding author upon a reasonable request.

## Supplementary Materials

The following supporting information can be downloaded at https://www.mdpi.com/article/10.3390/plants14233563/s1; File S1: T-TEST results for pairwise comparisons of the core (LT-AG-PUR, LT-AG-SIM and LT-PA-DRU, LT-PA-IGN for *A. glutinosa* and *P. abies*, respectively) and peripheral (GR-AG-01, GR-AG-02 and GR-PA-07, GR-PA-08 for *A. glutinosa* and *P. abies*, respectively) population genetic parameters of SAS software; File S2: The most probable number of clusters for *A. glutinosa* and *P. abies* using the Δ*K* criterion, as calculated by STRUCTURE analysis software; File S3: Primer and locus information.

## Abbreviations

The following abbreviations are used in this manuscript:

CPH	Central-periphery hypothesis
EST	Expressed sequence tag
GCU	Genetic conservation unit
PCoA	Principal coordinate analysis
PCR	Polymerase chain reaction
SSR	Simple sequence repeats

## References

[1] Kelleher C.T., De Vries S.M., Baliuckas V., Bozzano M., Frýdl J., Gonzalez Goicoechea P., Ivankovic M., Kandemir G., Koskela J., Kozioł C. (2015). Approaches to the Conservation of Forest Genetic Resources in Europe in the Context of Climate Change.

[2] Fady B., Aravanopoulos F.A., Alizoti P., Mátyás C., von Wühlisch G., Westergren M., Belletti P., Cvjetkovic B., Ducci F., Huber G. (2016). Evolution-based approach needed for the conservation and silviculture of peripheral forest tree populations. For. Ecol. Manag..

[3] Hampe A., Petit R.J. (2005). Conserving biodiversity under climate change: The rear edge matters. Mol. Ecol..

[4] Pörtner H.O., Roberts D.C., Tignor M., Poloczanska E.S., Mintenbeck K., Alegría A., Craig M., Langsdorf S., Löschke S., Möller V. (2022). Climate Change 2022: Impacts, Adaptation, and Vulnerability. Contribution of Working Group II to the Sixth Assessment Report of the Intergovernmental Panel on Climate Change.

[5] Mayr E. (1970). Populations, Species, and Evolution. An Abridgment of Animal Species and Evolution.

[6] Eckert C.G., Samis K.E., Lougheed S.C. (2008). Genetic variation across species’ geographical ranges: The central-marginal hypothesis and beyond. Mol. Ecol..

[7] Rajora O.P., DeVerno L., Mosseler A., Innes D.J. (1998). Genetic diversity and population structure of disjunct Newfoundland and central Ontario populations of eastern white pine (*Pinus strobus*). Can. J. Bot..

[8] Yang A., Dick C.W., Yao X., Huang H. (2016). Impacts of biogeographic history and marginal population genetics on species range limits: A case study of *Liriodendron chinense*. Sci. Rep..

[9] Jaramillo-Correa J.P., Bagnoli F., Grivet D., Fady B., Aravanopoulos F.A., Vendramin G.G., González-Martínez S.C. (2020). Evolutionary rate and genetic load in an emblematic Mediterranean tree following an ancient and prolonged population collapse. Mol. Ecol..

[10] Vucetich J.A., Waite T.A. (2003). Spatial patterns of demography and genetic processes across the species’ range: Null hypotheses forlandscape conservation genetics. Conserv. Genet..

[11] Milesi P., Kastally C., Dauphin B., Cervantes S., Bagnoli F., Budde K.B., Cavers S., Fady B., Faivre-Rampant P., González-Martínez S.C. (2024). Resilience of genetic diversity in forest trees over the Quaternary. Nat. Commun..

[12] Ha Y.H., An J.B., Chung J., Yoon J.W., Gil H.Y. (2025). Genotyping-by-sequencing reveals low genetic diversity and peripheral isolation in Southern populations of *Sophora koreensis*, a Korean endemic shrub. Sci. Rep..

[13] Svenning J.C., Skov F. (2007). Could the tree diversity pattern in Europe be generated by postglacial dispersal limitation?. Ecol. Lett..

[14] Feurdean A., Bhagwat S.A., Willis K.J., Birks H.J.B., Lischke H., Hickler T. (2013). Tree migration-rates: Narrowing the gap between inferred post-glacial rates and projected rates. PLoS ONE.

[15] Zhang Y., Li Z., Chen Q., Wang Y., Wang S., Wang G., Li P., Liu H., Li P., Xu C. (2025). Phylogeography and Population Demography of *Parrotia subaequalis*, a Hamamelidaceous Tertiary Relict ‘Living Fossil’Tree Endemic to East Asia Refugia: Implications from Molecular Data and Ecological Niche Modeling. Plants.

[16] Stamford M.D., Taylor E.B. (2004). Phylogeographic lineages of Arctic grayling (*Thymallus arcticus*) in North America, divergence, origins and affinities with Eurasian *Thymallus*. Mol. Ecol..

[17] Muller M.H., Leppala J., Savolainen O. (2008). Genome-wide effects of postglacial colonization in *Arabidopsis lyrata*. Heredity.

[18] Havrdová A., Douda J., Krak K., Vít P., Hadincová V., Zákravský P., Mandák B. (2015). Higher genetic diversity in recolonized areas than in refugia of *Alnus glutinosa* triggered by continent—Wide lineage admixture. Mol. Ecol..

[19] McVean D.N. (1953). Biological flora of the British Isles: *Alnus* Mill. *Alnus glutinosa* (L.) *Gaertn*. (*A*. *rotundifolia Stokes*). J. Ecol..

[20] Douda J., Čejková A., Douda K., Kochánková J. (2009). Development of alder carr after the abandonment of wet grasslands during the last 70 years. Ann. For. Sci..

[21] Douda J. (2010). The role of landscape configuration in plant composition of floodplain forests across different physiographic areas. J. Veg. Sci..

[22] Cox K., Vanden Broeck A., Van Calster H., Mergeay J. (2011). Temperature—Related natural selection in a wind—Pollinated tree across regional and continental scales. Mol. Ecol..

[23] Lepais O., Muller S.D., Ben Saad-Limam S., Benslama M., Rhazi L., Belouahem-Abed D., Daoud-Bouattour A., Gammar A.M., Ghrabi-Gammar Z., Bacles C.F.E. (2013). High genetic diversity and distinctiveness of rear-edge climate relicts maintained by ancient tetraploidisation for *Alnus glutinosa*. PLoS ONE.

[24] Beatty G.E., Montgomery W.I., Tosh D.G., Provan J. (2015). Genetic provenance and best practice woodland management: A case study in native alder (*Alnus glutinosa*). Tree Genet. Genomes.

[25] Mandák B., Vít P., Krak K., Trávníček P., Havrdová A., Hadincová V., Zákravský P., Jarolímová V., Bacles C.F.E., Douda J. (2016). Flow cytometry, microsatellites and niche models reveal the origins and geographical structure of *Alnus glutinosa* populations in Europe. Ann. Bot..

[26] Mingeot D., Husson C., Mertens P., Watillon B., Bertin P., Druart P. (2016). Genetic diversity and genetic structure of black alder (*Alnus glutinosa* [L.] Gaertn) in the Belgium-Luxembourg-France cross-border area. Tree Genet. Genomes.

[27] Verbylaitė R., Aravanopoulos F.A., Baliuckas V., Juškauskaitė A. (2023). Genetic monitoring of *Alnus glutinosa* natural populations using two generation cohorts. Forests.

[28] Caudullo G., Tinner W., De Rigo D., San-Miguel-Ayanz J., de Rigo D., Caudullo G., Houston Durrant T., Mauri A. (2016). Picea abies in Europe: Distribution, habitat, usage and threats. European Atlas of Forest Tree Species.

[29] Jalas J., Suominen J. (1973). Atlas Florae Europaeae: Distribution of Vascular Plants in Europe—Vol. 2: Gymnospermae (Pinaceae To Ephedraceae).

[30] Farjon A., Filer D. (2013). An Atlas of the World’s Conifers: An Analysis of Their Distribution, Biogeography, Diversity and Conservation Status.

[31] Horgan T., Keane M., McCarthy R., Lally M., Thompson D., O’Carroll J. (2003). A Guide to Forest Tree Species Selection and Silviculture in IRELAND.

[32] Tjoelker M.G., Boratynski A., Bugala W. (2007). Biology and Ecology of Norway Spruce.

[33] Caré O., Müller M., Vornam B., Höltken A.M., Kahlert K., Krutovsky K.V., Gailing O., Leinemann L. (2018). High morphological differentiation in crown architecture contrasts with low population genetic structure of German Norway spruce stands. Forests.

[34] Westergren M., Bozic G., Kraigher H. (2018). Genetic diversity of core vs. peripheral Norway spruce native populations at a local scale in Slovenia. IForest.

[35] Stojnić S., Avramidou E.V., Fussi B., Westergren M., Orlović S., Matović B., Trudić B., Kraigher H., Aravanopoulos F.A., Konnert M. (2019). Assessment of genetic diversity and population genetic structure of Norway spruce (*Picea abies* (L.) Karsten) at its southern lineage in Europe. Implications for conservation of forest genetic resources. Forests.

[36] Wang X., Bernhardsson C., Ingvarsson P.K. (2020). Demography and natural selection have shaped genetic variation in the widely distributed conifer Norway spruce (*Picea abies*). Genome Biol. Evol..

[37] Unger G.M., Konrad H., Geburek T. (2011). Does spatial genetic structure increase with altitude? An answer from *Picea abies* in Tyrol, Austria. Plant Syst. Evol..

[38] Verbylaitė R., Pliūra A., Lygis V., Suchockas V., Jankauskienė J., Labokas J. (2017). Genetic diversity and its spatial distribution in self-regenerating Norway spruce and Scots pine stands. Forests.

[39] Máchová P., Trčková O., Cvrčková H. (2018). Use of nuclear microsatellite loci for evaluating genetic diversity of selected populations of *Picea abies* (L.) Karsten in the Czech Republic. Forests.

[40] Bínová Z., Korecký J., Dvořák J., Bílý J., Zádrapová D., Jansa V., Lstibůrek M. (2020). Genetic structure of Norway spruce ecotypes studied by SSR markers. Forests.

[41] Tollefsrud M.M., Kissling R.O.Y., Gugerli F., Johnsen Ø., Skrøppa T., Cheddadi R., VAN DER Knaap W.O., Latałowa M., Terhürne-Berson R., Litt T. (2008). Genetic consequences of glacial survival and postglacial colonization in Norway spruce: Combined analysis of mitochondrial DNA and fossil pollen. Mol. Ecol..

[42] Evanno A., Regnaut S., Goudet J. (2005). Detecting the number of clusters of individuals using the software structure: A simulation study. Mol. Ecol..

[43] Konnert M., Fady B., Gömöry D., A’Hara S., Wolter F., Ducci F., Koskela J., Bozzano M., Maaten T., Kowalczyk J. (2015). European Forest Genetic Resources Programme (EUFORGEN): Use and Transfer of Forest Reproductive Material in Europe in the Context of Climate Change.

[44] Cvjetkovic B., Konnert M., Fussi B., Mataruga M., Sijacic-Nikolic M., Danicic V., Lucic A. (2017). Norway spruce (*Picea abies* Karst.) variability in progeny tests in Bosnia and Herzegovina. Genetika.

[45] Whitlock M.C. (2011). G’_ST_ and D do not replace F_ST_. Mol. Ecol..

[46] Piry S., Luikart G., Cornuet J. (1999). Bottleneck: A computer program for detecting recent reductions in the effective population size using allele frequency data. J. Hered..

[47] Ganopoulos I., Aravanopoulos F.A., Argiriou A., Kalivas A., Tsaftaris A. (2011). Is the genetic diversity of small scattered forest tree populations at the southern limits of their range more prone to stochastic events? A wild cherry case study by microsatellite-based markers. Tree Genet. Genomes.

[48] Picard N., Marchi M., Serra-Varela M.J., Westergren M., Cavers S., Notivol E., Piotti A., Alizoti P., Bozzano M., González-Martínez S.C. (2022). Marginality indices for biodiversity conservation in forest trees. Ecol. Indic..

[49] Avtzis D.N., Aravanopoulos F.A. (2011). Host tree and insect genetic diversity on the borderline of natural distribution: A case study of *Picea abies* and *Pityogenes chalcographus* (Coleoptera, Scolytinae) in Greece. Silva Fenn..

[50] Koskela J., Lefèvre F., Schueler S., Kraigher H., Olrik D.C., Hubert J., Longauer R., Bozzano M., Yrjänä L., Alizoti P. (2013). Translating conservation genetics into management: Pan-European minimum requirements for dynamic conservation units of forest tree genetic diversity. Biol. Conserv..

[51] De Vries S.M., Alan M., Bozzano M., Burianek V., Collin E., Cottrell J., Ivankovic M., Kelleher C.T., Koskela J., Rotach P. (2015). Pan-European strategy for Genetic Conservation of Forest Trees and Establishment of a Core Network of Dynamic Conservation Units.

[52] (2009). Distribution Map of Black Alder (*Alnus glutinosa*) EUFORGEN. https://www.euforgen.org/fileadmin/templates/euforgen.org/upload/Documents/Maps/PDF/Alnus_glutinosa.pdf.

[53] Caudullo G., Welk E., San-Miguel-Ayanz J. (2017). Distribution map of Norway spruce (*Picea abies*). Chorological maps for the main European woody species. Data Brief.

[54] Wu B., Lian C., Hogetsu T. (2002). Development of microsatellite markers in white birch (*Betula platyphylla* var. japonica). Mol. Ecol..

[55] Gürcan K., Mehlenbacher S.A., Botta R., Boccacci P. (2010). Development, characterization, segregation, and mapping of microsatellite markers for European hazelnut (*Corylus avellana* L.) from enriched genomic libraries and usefulness in genetic diversity studies. Tree Genet. Genomes.

[56] Kulju K.K., Pekkinen M., Varvio S. (2004). Twenty-three microsatellite primer pairs for *Betula pendula* (Betulaceae). Mol. Ecol. Notes.

[57] Tsuda Y., Ueno S., Ide Y., Tsumura Y. (2009). Development of 14 EST-SSRs for *Betula maximowicziana* and their applicability to related species. Conserv. Genet..

[58] Ogyu K., Tsuda Y., Sugaya T., Yoshimaru H., Ide Y. (2003). Identification and characterization of microsatellite loci in *Betula maximowicziana* Regel. Mol. Ecol. Notes.

[59] Tsuda Y., Ueno S., Ranta J., Salminen K., Ide Y., Shinohara K., Tsumura Y. (2009). Development of 11 EST-SSRs for Japanese white birch, *Betula platyphylla* var. japonica and their transferability to related species. Conserv. Genet..

[60] Lepais O., Bacles C.F. (2011). De novo discovery and multiplexed amplification of microsatellite markers for black alder (*Alnus glutinosa*) and related species using SSR-enriched shotgun pyrosequencing. J. Hered..

[61] Drašnarová A., Krak K., Vít P., Doudová J., Douda J., Hadincová V., Zákravský P., Mandák B. (2014). Cross-amplification and multiplexing of SSR markers for *Alnus glutinosa* and *A. incana*. Tree Genet. Genomes.

[62] Besnard G., Acheré V., Faivre Rampant P., Favre J.M., Jeandroz S. (2003). A set of cross—Species amplifying microsatellite markers developed from DNA sequence databanks in *Picea* (Pinaceae). Mol. Ecol. Notes.

[63] Schubert R., Mueller-Starck G., Riegel R. (2001). Development of EST-PCR markers and monitoring their intrapopulational genetic variation in *Picea abies* (L.) *Karst*. Theor. Appl. Genet..

[64] Rungis D., Bérubé Y., Zhang J., Ralph S., Ritland C.E., Ellis B.E., Douglas C., Ritland K., Bérubé Y., Bohlmann J. (2004). Robust simple sequence repeat markers for spruce (*Picea* spp.) from expressed sequence tags. Theor. Appl. Genet..

[65] Pfeiffer A., Olivieri A.M., Morgante M. (1997). Identification and characterization of microsatellites in Norway spruce (*Picea abies* K.). Genome.

[66] Hodgetts R.B., Aleksiuk M.A., Brown A., Clarke C., Macdonald E., Nadeem S., Khasa D. (2001). Development of microsatellite markers for white spruce (*Picea glauca*) and related species. Theor. Appl. Genet..

[67] Scotti I., Paglia G., Magni F., Morgante M. (2002). Efficient development of dinucleotide microsatellite markers in Norway spruce (*Picea abies* Karst.) through dot-blot selection. Theor. Appl. Genet..

[68] Scotti I., Magni F., Paglia G., Morgante M. (2002). Trinucleotide microsatellites in Norway spruce *(Picea abies*): Their features and the development of molecular markers. Theor. Appl. Genet..

[69] Scotti I., Magni F., Fink R., Powell W., Binelli G., Hedley P.E. (2000). Microsatellite repeats are not randomly distributed within Norway spruce (*Picea abies* K.) expressed sequences. Genome.

[70] Rajora O.P., Rahman M.H., Dayanandan S., Mosseler A. (2001). Isolation, characterization, inheritance and linkage of microsatellite DNA markers in white spruce *(Picea glauca*) and their usefulness in other spruce species. Mol. Genet. Genom..

[71] Scotti I., Paglia G., Magni F., Morgante M. (2006). Population genetics of Norway spruce (*Picea abies* Karst.) at regional scale: Sensitivity of different microsatellite motif classes in detecting differentiation. Ann. For. Sci..

[72] Van Oosterhout C., Hutchinson W.F., Wills D.P., Shipley P. (2004). Micro—Checker: Software for identifying and correcting genotyping errors in microsatellite data. Mol. Ecol. Notes.

[73] Peakall R.O., Smouse P.E. (2006). GENALEX 6: Genetic analysis in Excel. Population genetic software for teaching and research. Mol. Ecol. Notes.

[74] Peakall R.O., Smouse P.E. (2012). GenAlEx 6.5: Genetic analysis in Excel. Population genetic software for teaching and research—An update. Bioinformatics.

[75] Smouse P.E., Banks S.C., Peakall R. (2017). Converting quadratic entropy to diversity: Both animals and alleles are diverse, but some are more diverse than others. PLoS ONE.

[76] Goudet J. (1995). FSTAT (Version 1.2): A computer program to calculate F-statistics. J. Hered..

[77] Waples R.S., Do C.H. (2010). Linkage disequilibrium estimates of contemporary *N*_e_ using highly variable genetic markers: A largely untapped resource for applied conservation and evolution. Evol. Appl..

[78] Do C., Waples R.S., Peel D., Macbeth G.M., Tillett B.J., Ovenden J.R. (2014). NeEstimator v2: Re-implementation of software for the estimation of contemporary effective population size (Ne) from genetic data. Mol. Ecol. Resour..

[79] Meirmans P.G., Hedrick P.W. (2011). Assessing population structure: *F*_ST_ and related measures. Mol. Ecol. Resour..

[80] Pritchard J.K., Stephens M., Donnelly P. (2000). Inference of population structure using multilocus genotype data. Genetics.

[81] Falush D., Stephens M., Pritchard J.K. (2003). Inference of population structure using multilocus genotype data: Linked loci and correlated allele frequencies. Genetics.

[82] Falush D., Matthew S., Pritchard J.K. (2007). Inference of population structure using multilocus genotype data: Dominant markers and null alleles. Mol. Ecol. Notes.

[83] Hubisz M.J., Falush D., Stephens M., Pritchard J.K. (2009). Inferring weak population structure with the assistance of sample group information. Mol. Ecol. Resour..

[84] Li Y.L., Liu J.X. (2018). StructureSelector: A web based software to select and visualize the optimal number of clusters using multiple methods. Mol. Ecol. Resour..

[85] Aravanopoulos F.A. (2016). Conservation and monitoring of tree genetic resources in temperate forests. Curr. For. Rep..

