# Supplementary material for: The Central-Periphery Hypothesis Revisited: Implications for Long-Term Genetic Conservation
Source: Plants (Basel). 2025 Nov 21;14(23):3563. doi: 10.3390/plants14233563 (PMC12693884; doi:10.3390/plants14233563)

Supplement S2.

The Central-Periphery Hypothesis Revisited: Implications for Long-Term Genetic Conservation

Figure S1. The probability of likelihood for the number of clusters for *Alnus glutinosa*, as determined by the ΔK criterion

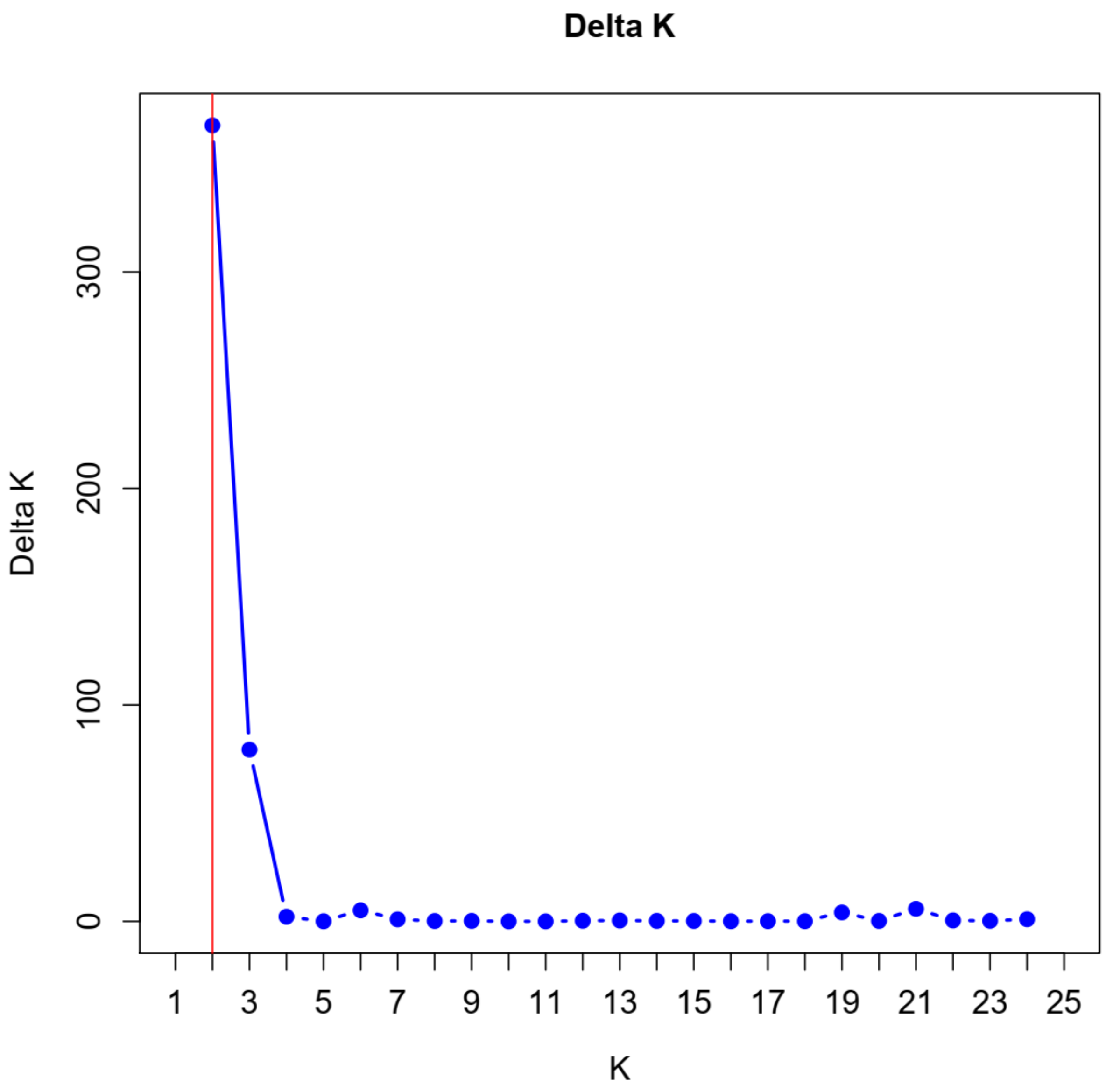

Figure S2. The probability of likelihood for the number of clusters for *Picea abies,* as determined by the ΔK criterion

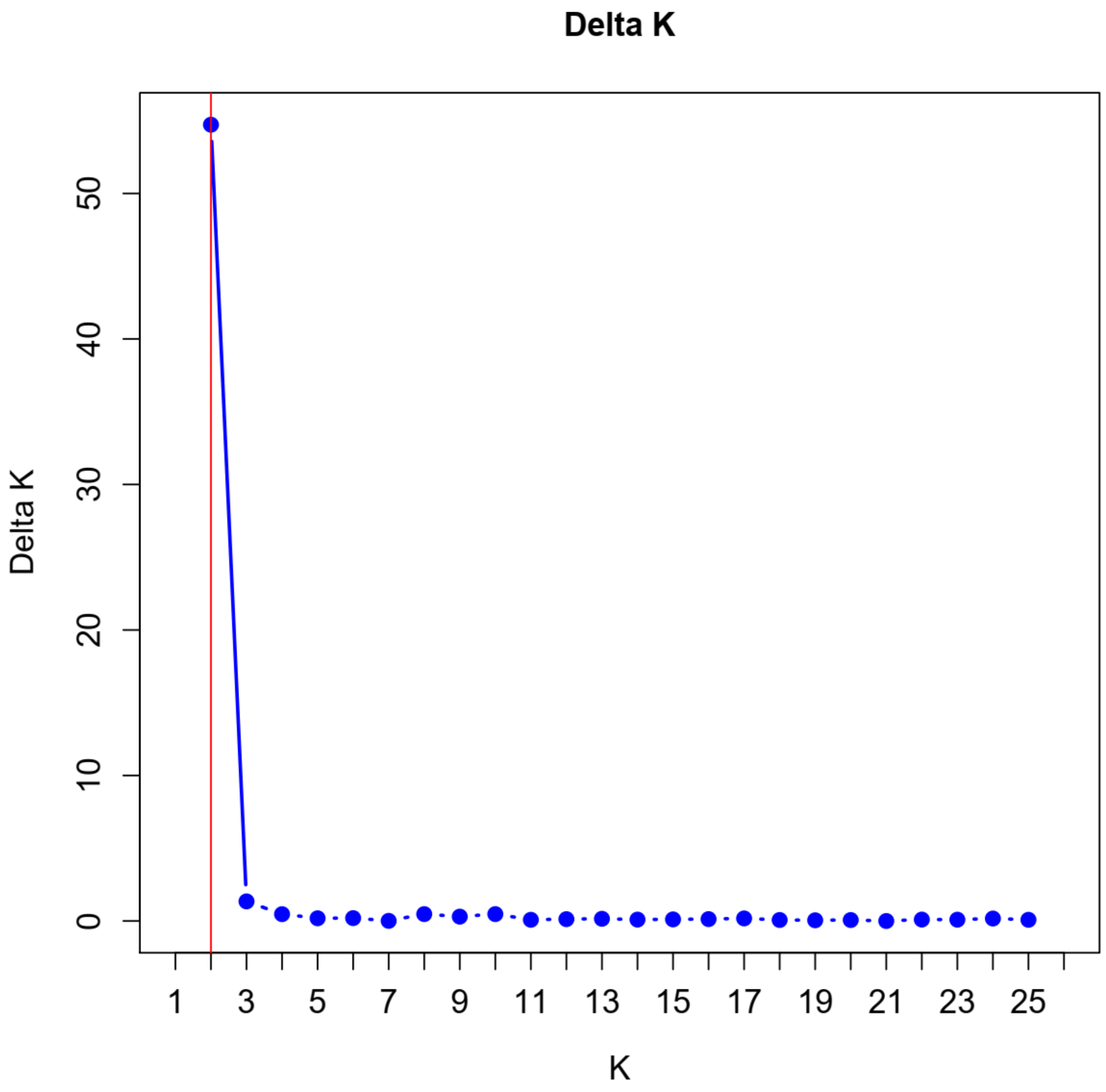

Supplement: Supplementary file 1 [file plants-14-03563-s001.zip › File S2.pdf]
