# Supplementary material for: The Central-Periphery Hypothesis Revisited: Implications for Long-Term Genetic Conservation
Source: Plants (Basel). 2025 Nov 21;14(23):3563. doi: 10.3390/plants14233563 (PMC12693884; doi:10.3390/plants14233563)
Supplement: Supplementary file 1 [file plants-14-03563-s001.zip › File S1.pdf]

Supplement S1.

The Central-Periphery Hypothesis Revisited: Implications for Long-Term Genetic Conservation

T-test results for peripheral versus core population genetic variables.

Alnus glutinosa

The SAS System

The TTEST Procedure

Table S1. Variable: Na

pops	N	Mean	Std Dev	Std Err	Minimum	Maximum
**GR**	18	11.1111	4.0131	0.9459	5.0000	22.0000
**LT**	18	11.5556	3.2760	0.7722	4.0000	16.0000
**Diff (1-2)**		-0.4444	3.6631	1.2210		

pops	Method	Mean	95% CL Mean		Std Dev	95% CL Std Dev	
**GR**		11.1111	9.1155	13.1068	4.0131	3.0113	6.0161
**LT**		11.5556	9.9265	13.1847	3.2760	2.4583	4.9112
**Diff (1-2)**	**Pooled**	-0.4444	-2.9259	2.0370	3.6631	2.9630	4.7994
**Diff (1-2)**	**Satterthwaite**	-0.4444	-2.9296	2.0407			

Method	Variances	DF	t Value	Pr > |t|
**Pooled**	Equal	34	-0.36	0.7181
**Satterthwaite**	Unequal	32.69	-0.36	0.7182

Equality of Variances

Method	Num DF	Den DF	F Value	Pr > F
**Folded F**	17	17	1.50	0.4112

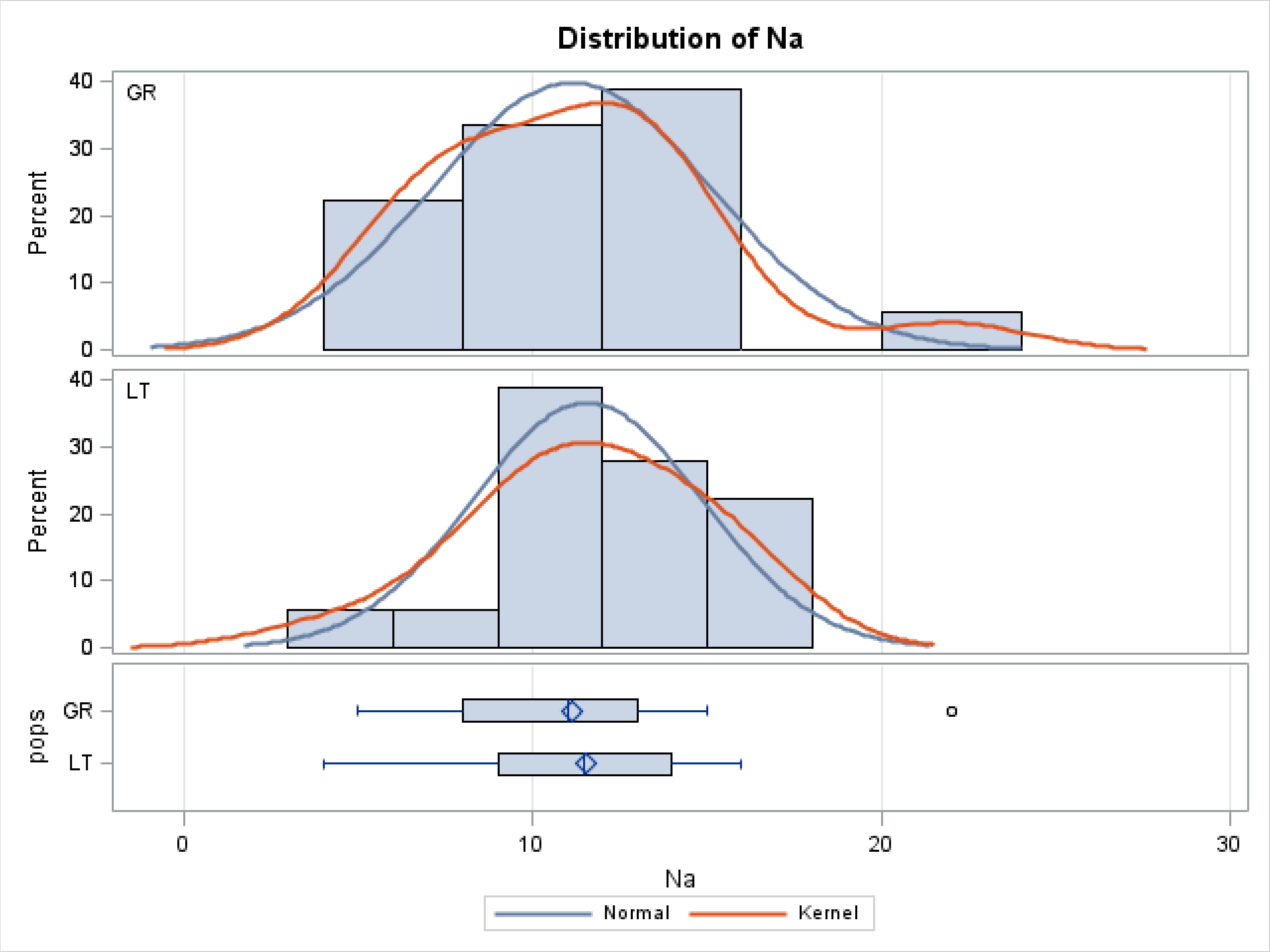
Distribution of Na
GR
LT
Percent
0
10
20
30
40
pops
Na
Normal
Kernel

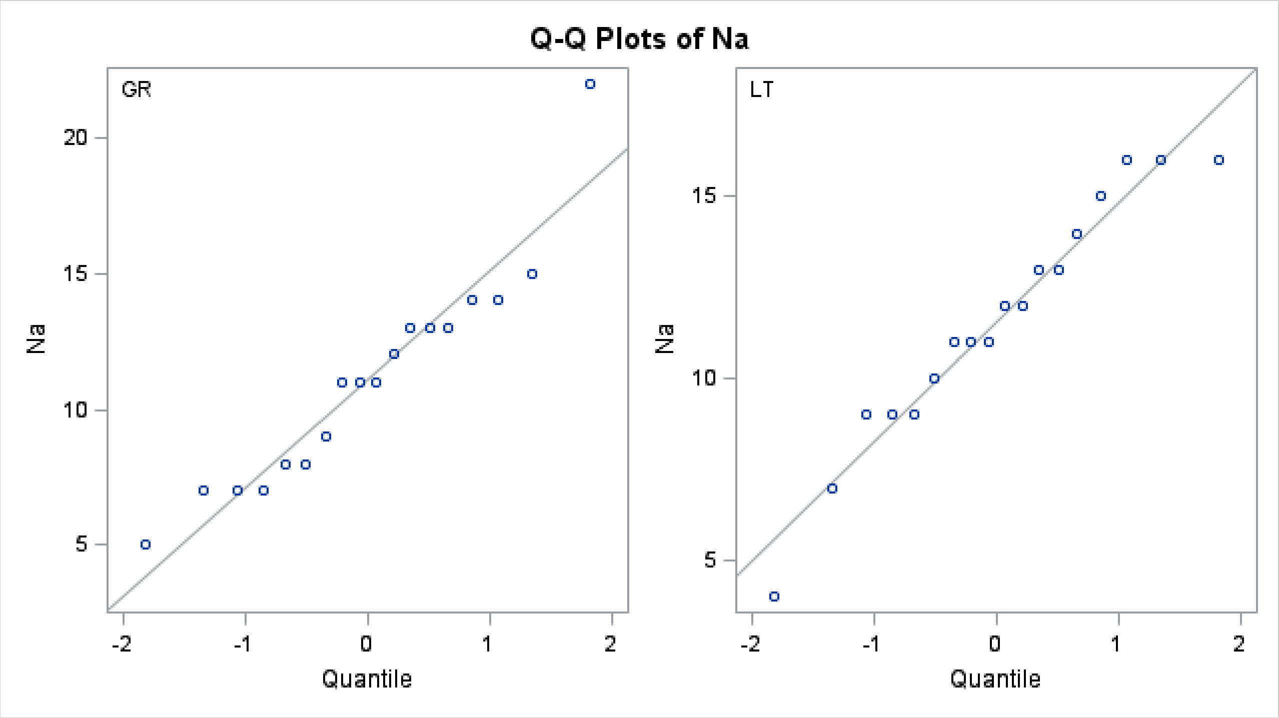
Q-Q Plots of Na
GR
LT
Na
Quantile

Table S2. Variable: Ne

pops	N	Mean	Std Dev	Std Err	Minimum	Maximum
**GR**	18	4.7875	2.0705	0.4880	1.5140	9.4340
**LT**	18	5.0494	2.2942	0.5408	1.6140	9.5810
**Diff (1-2)**		-0.2619	2.1852	0.7284		

pops	Method	Mean	95% CL Mean		Std Dev	95% CL Std Dev	
**GR**		4.7875	3.7579	5.8171	2.0705	1.5537	3.1040
**LT**		5.0494	3.9085	6.1903	2.2942	1.7216	3.4394
**Diff (1-2)**	**Pooled**	-0.2619	-1.7422	1.2184	2.1852	1.7676	2.8631
**Diff (1-2)**	**Satterthwaite**	-0.2619	-1.7428	1.2189			

Method	Variances	DF	t Value	Pr > |t|
**Pooled**	Equal	34	-0.36	0.7214
**Satterthwaite**	Unequal	33.648	-0.36	0.7214

Equality of Variances

Method	Num DF	Den DF	F Value	Pr > F
**Folded F**	17	17	1.23	0.6770

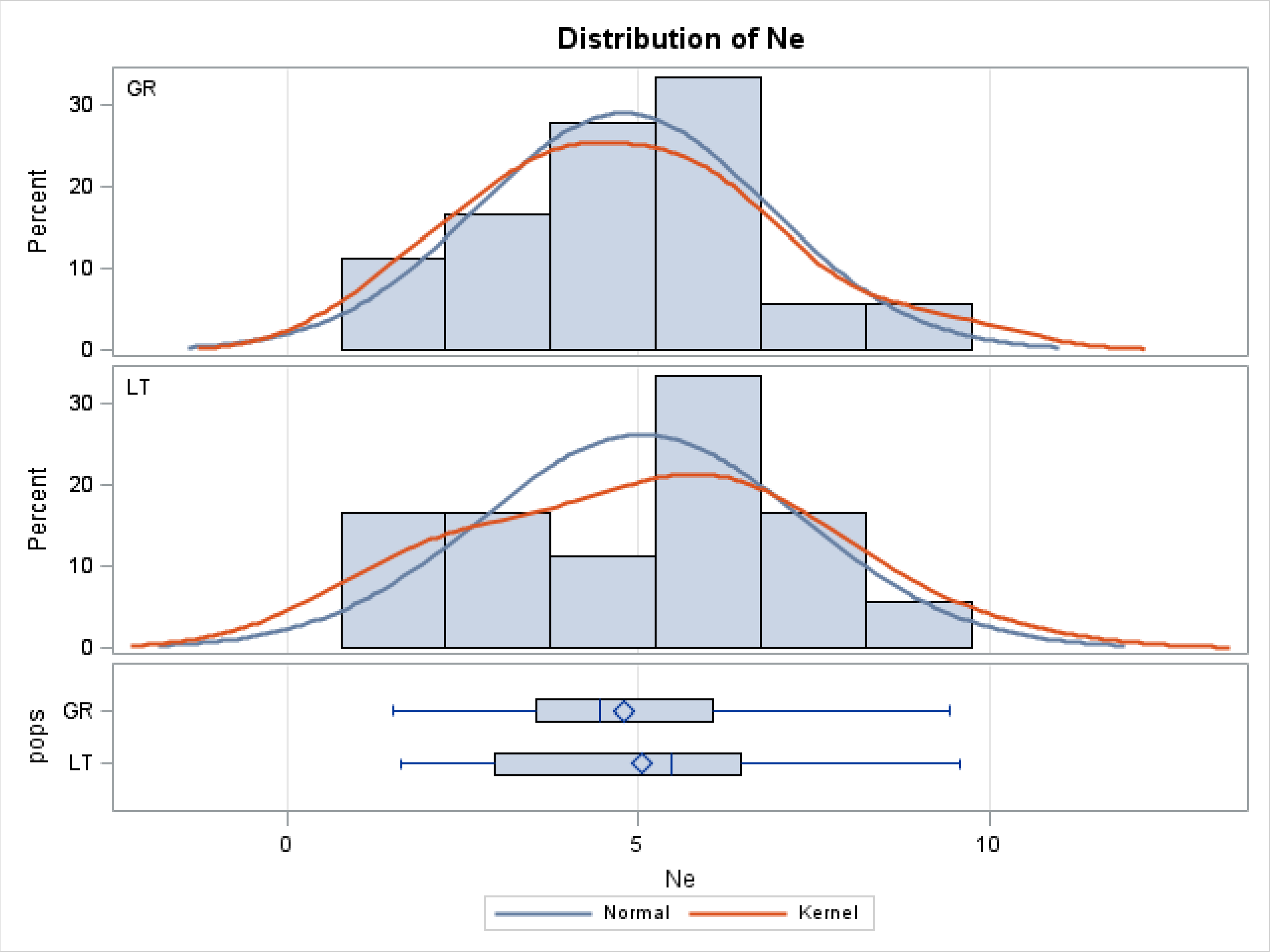
Distribution of Ne
GR
LT
Percent
0
10
20
30
pops
Ne
0
5
10
Normal
Kernel

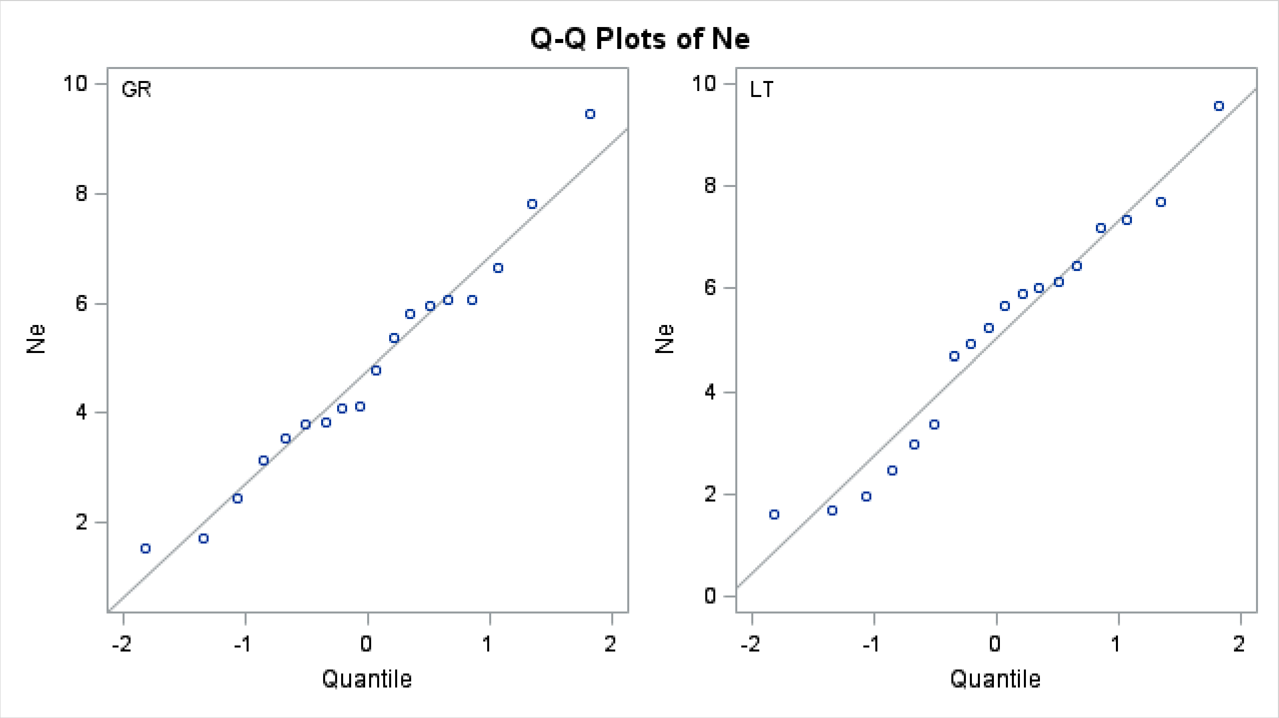
Q-Q Plots of Ne
GR
LT
Ne
Quantile
-2
-1
0
1
2
0
2
4
6
8
10

Table S3. Variable: Ho

pops	N	Mean	Std Dev	Std Err	Minimum	Maximum
**GR**	18	0.8018	0.1684	0.0397	0.3730	1.0000
**LT**	18	0.7188	0.1664	0.0392	0.3880	0.9630
**Diff (1-2)**		0.0829	0.1674	0.0558		

pops	Method	Mean	95% CL Mean		Std Dev	95% CL Std Dev	
**GR**		0.8018	0.7180	0.8855	0.1684	0.1264	0.2525
**LT**		0.7188	0.6361	0.8016	0.1664	0.1248	0.2494
**Diff (1-2)**	**Pooled**	0.0829	-0.0305	0.1964	0.1674	0.1354	0.2193
**Diff (1-2)**	**Satterthwaite**	0.0829	-0.0305	0.1964			

Method	Variances	DF	t Value	Pr > |t|
**Pooled**	Equal	34	1.49	0.1464
**Satterthwaite**	Unequal	33.995	1.49	0.1464

Equality of Variances

Method	Num DF	Den DF	F Value	Pr > F
**Folded F**	17	17	1.03	0.9598

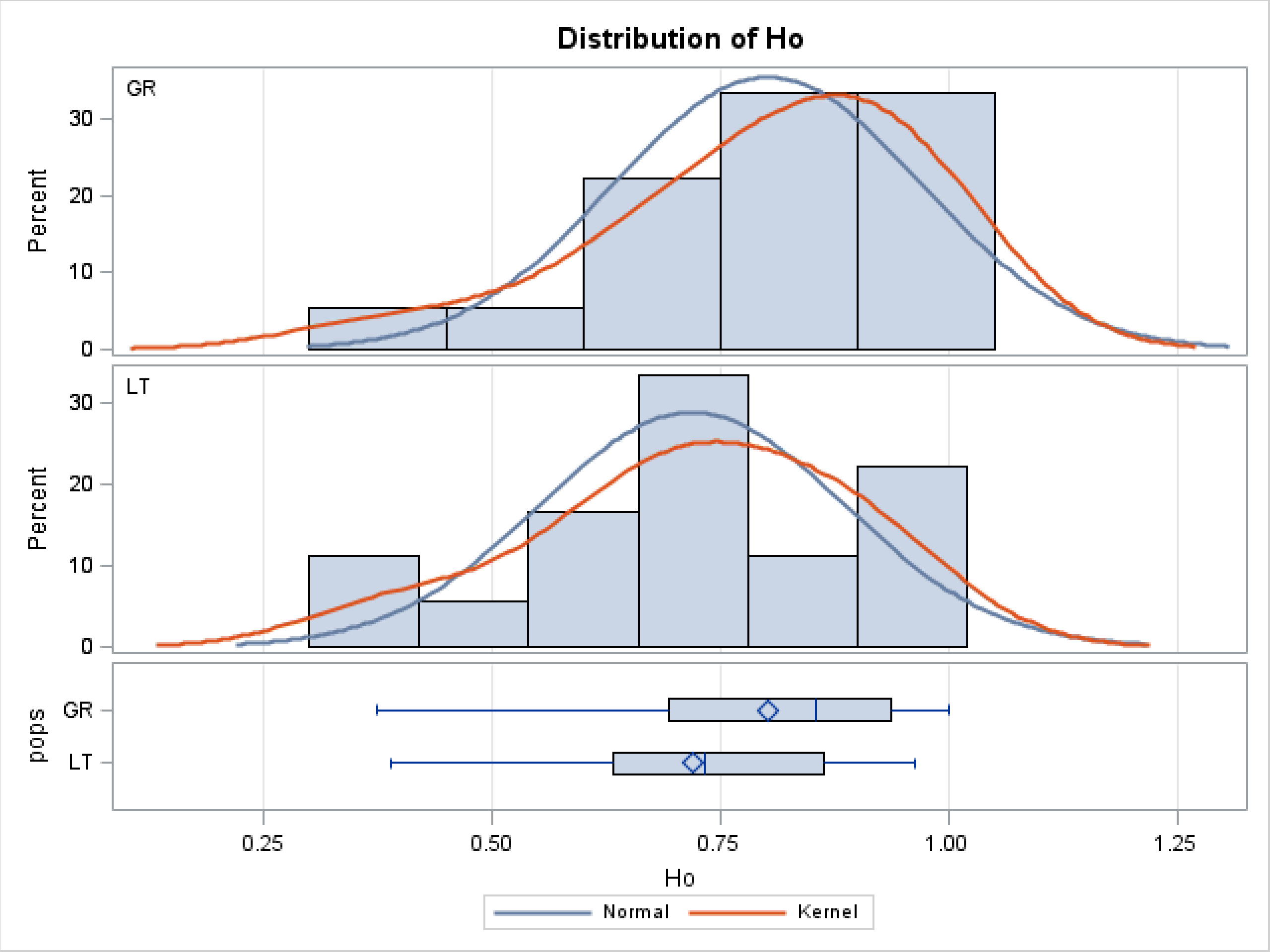
Distribution of Ho
GR
LT
Percent
pops
0
10
20
30
0.25
0.50
0.75
1.00
1.25
Ho
Normal
Kernel

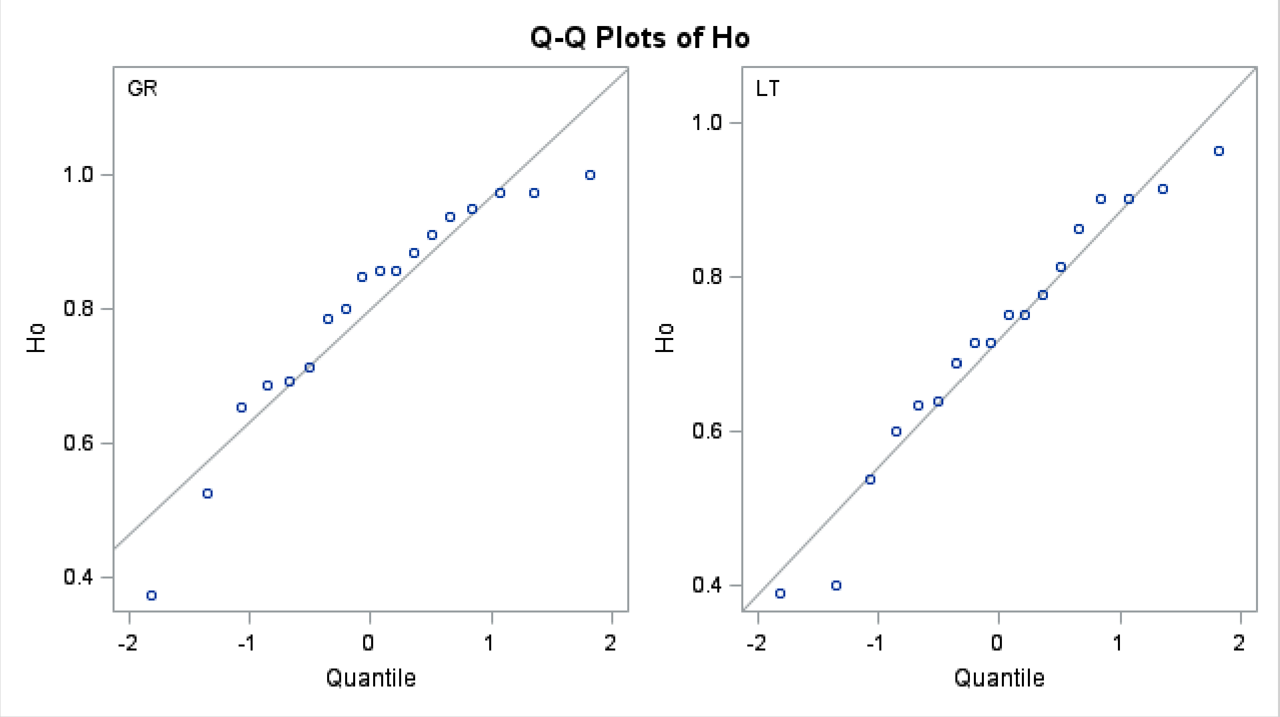
Q-Q Plots of Ho
GR
LT
Ho
Quantile
0.4
0.6
0.8
1.0
-2
-1
0
1
2

Table S4. Variable: He

pops	N	Mean	Std Dev	Std Err	Minimum	Maximum
**GR**	18	0.7383	0.1505	0.0355	0.3390	0.8940
**LT**	18	0.7383	0.1640	0.0387	0.3800	0.8960
**Diff (1-2)**		0.000056	0.1574	0.0525		

pops	Method	Mean	95% CL Mean		Std Dev	95% CL Std Dev	
**GR**		0.7383	0.6635	0.8132	0.1505	0.1130	0.2257
**LT**		0.7383	0.6567	0.8198	0.1640	0.1231	0.2459
**Diff (1-2)**	**Pooled**	0.000056	-0.1066	0.1067	0.1574	0.1273	0.2062
**Diff (1-2)**	**Satterthwaite**	0.000056	-0.1066	0.1067			

Method	Variances	DF	t Value	Pr > |t|
**Pooled**	Equal	34	0.00	0.9992
**Satterthwaite**	Unequal	33.753	0.00	0.9992

Equality of Variances

Method	Num DF	Den DF	F Value	Pr > F
**Folded F**	17	17	1.19	0.7276

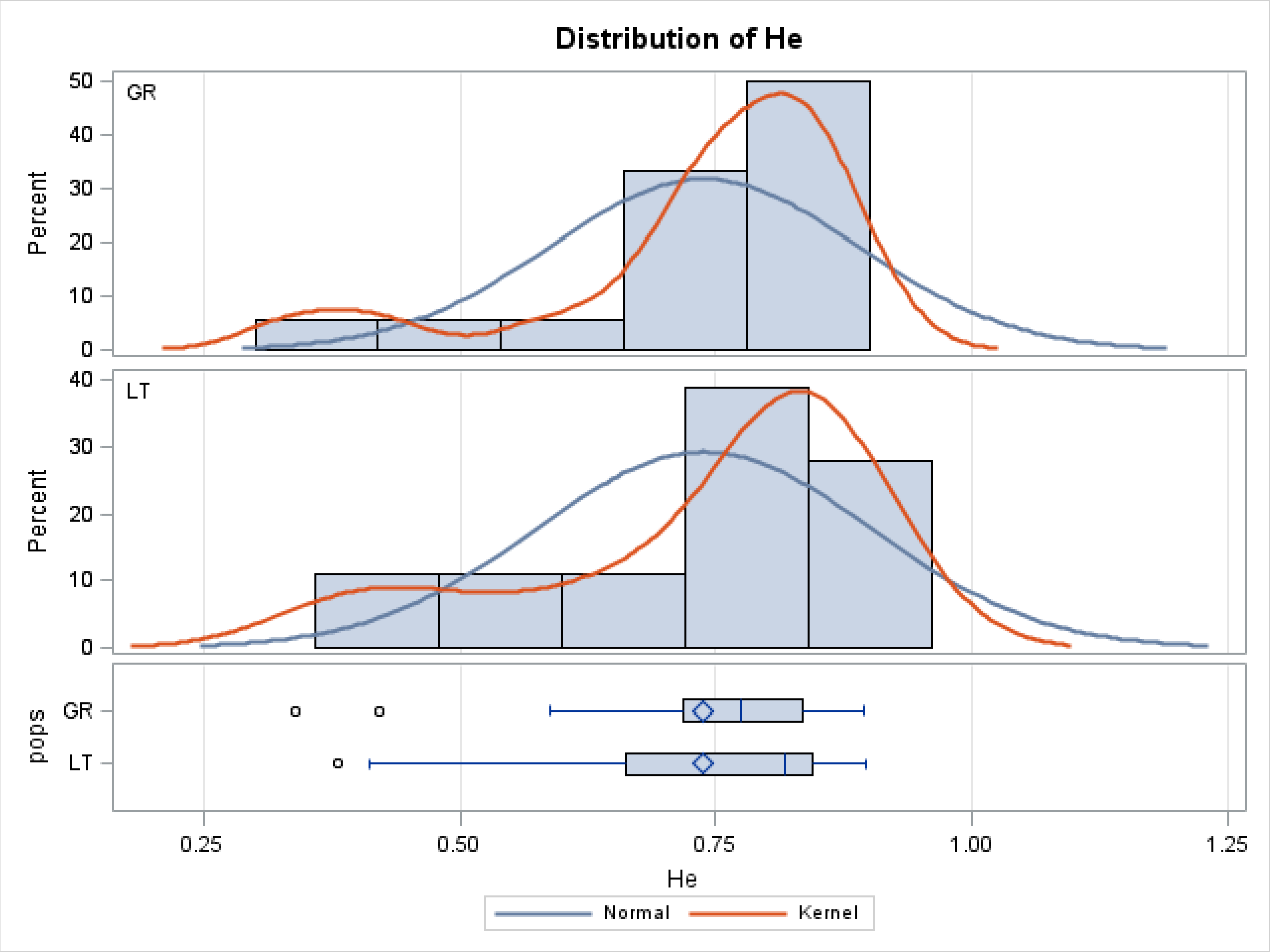
Distribution of He
GR
LT
Percent
pops
He
Normal
Kernel

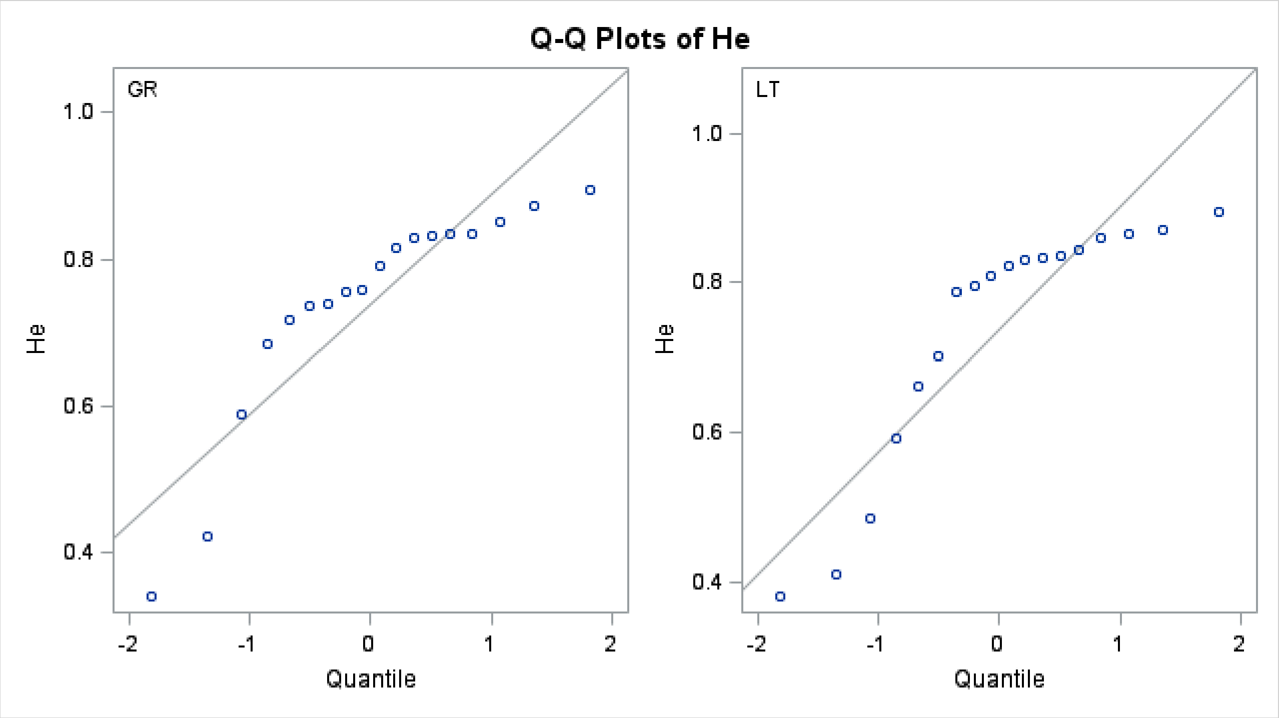
Q-Q Plots of He
GR
LT
He
Quantile

Table S5. Variable: Fis

pops	N	Mean	Std Dev	Std Err	Minimum	Maximum
**GR**	18	-0.1004	0.2156	0.0508	-0.4600	0.2300
**LT**	18	0.0242	0.1342	0.0316	-0.3020	0.2500
**Diff (1-2)**		-0.1246	0.1796	0.0599		

pops	Method	Mean	95% CL Mean		Std Dev	95% CL Std Dev	
**GR**		-0.1004	-0.2077	0.00677	0.2156	0.1618	0.3232
**LT**		0.0242	-0.0426	0.0909	0.1342	0.1007	0.2012
**Diff (1-2)**	**Pooled**	-0.1246	-0.2463	-0.00297	0.1796	0.1452	0.2353
**Diff (1-2)**	**Satterthwaite**	-0.1246	-0.2471	-0.00209			

Method	Variances	DF	t Value	Pr > |t|
**Pooled**	Equal	34	-2.08	0.0450
**Satterthwaite**	Unequal	28.454	-2.08	0.0465

Equality of Variances

Method	Num DF	Den DF	F Value	Pr > F
**Folded F**	17	17	2.58	0.0585

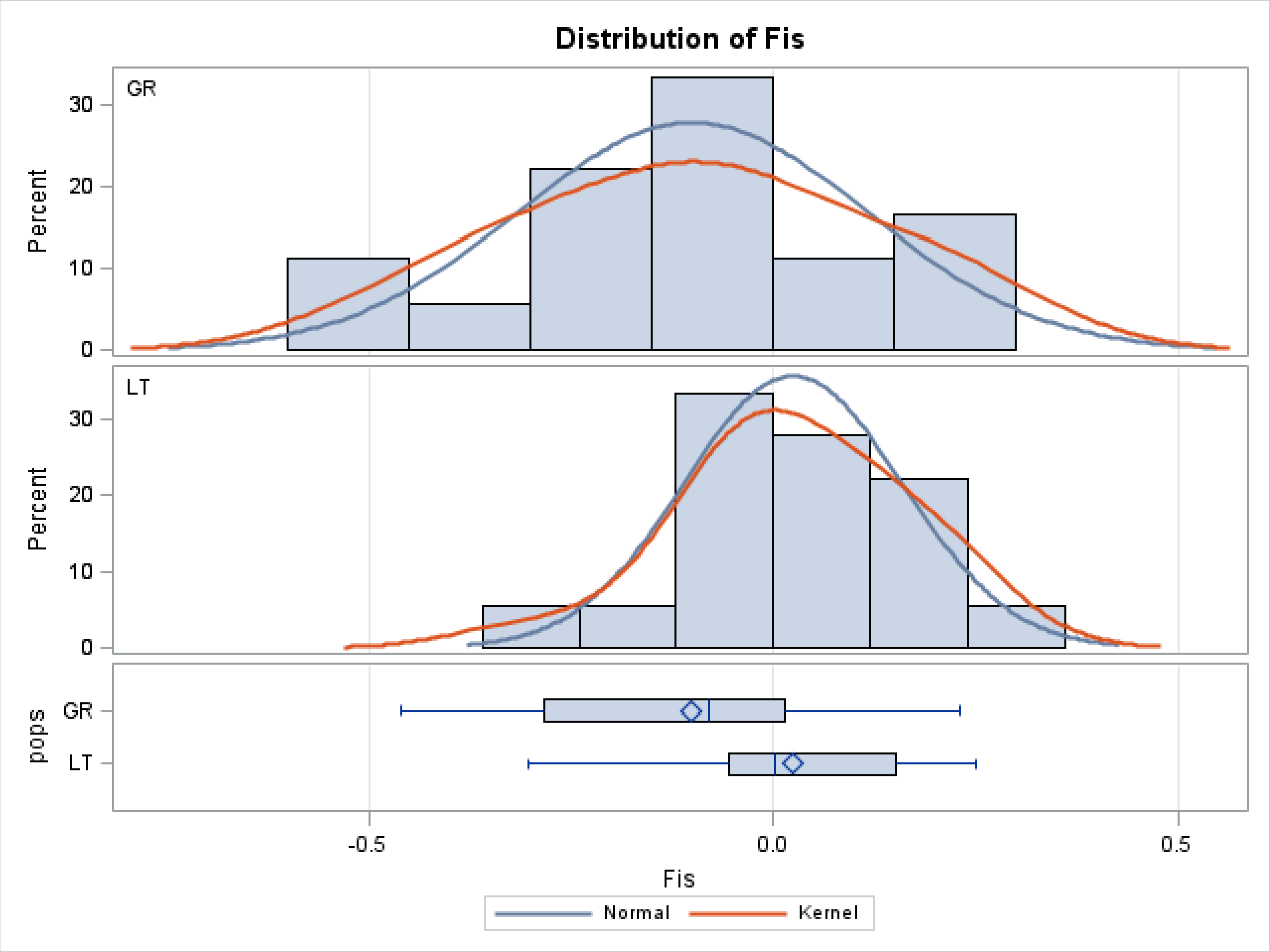
Distribution of Fis
GR
LT
Percent
pops
Fis
Normal
Kernel

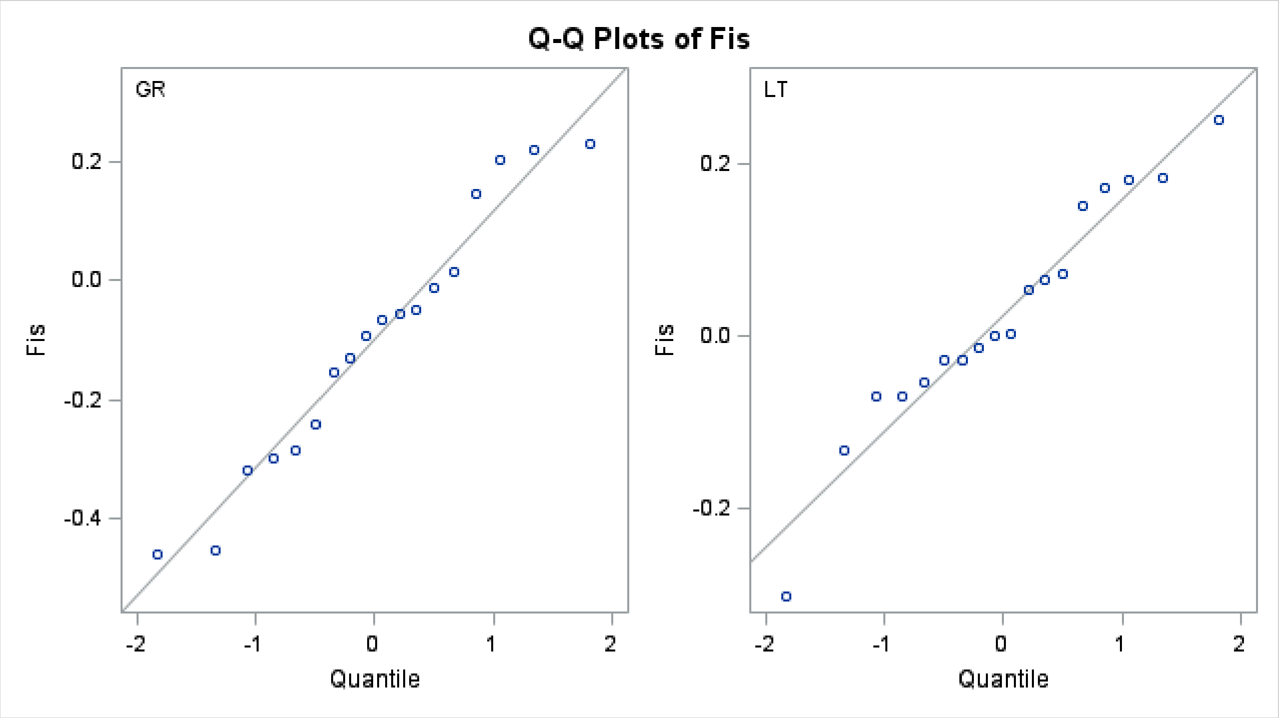
Q-Q Plots of Fis
GR
LT
Fis
Quantile

Table S6. Variable: Ar

pops	N	Mean	Std Dev	Std Err	Minimum	Maximum
**GR**	18	10.6764	3.8455	0.9064	4.6000	21.1830
**LT**	18	11.1494	3.1321	0.7382	3.9600	15.6290
**Diff (1-2)**		-0.4731	3.5070	1.1690		

pops	Method	Mean	95% CL Mean		Std Dev	95% CL Std Dev	
**GR**		10.6764	8.7641	12.5887	3.8455	2.8856	5.7650
**LT**		11.1494	9.5919	12.7070	3.1321	2.3503	4.6954
**Diff (1-2)**	**Pooled**	-0.4731	-2.8487	1.9026	3.5070	2.8367	4.5949
**Diff (1-2)**	**Satterthwaite**	-0.4731	-2.8523	1.9062			

Method	Variances	DF	t Value	Pr > |t|
**Pooled**	Equal	34	-0.40	0.6883
**Satterthwaite**	Unequal	32.662	-0.40	0.6884

Equality of Variances

Method	Num DF	Den DF	F Value	Pr > F
**Folded F**	17	17	1.51	0.4060

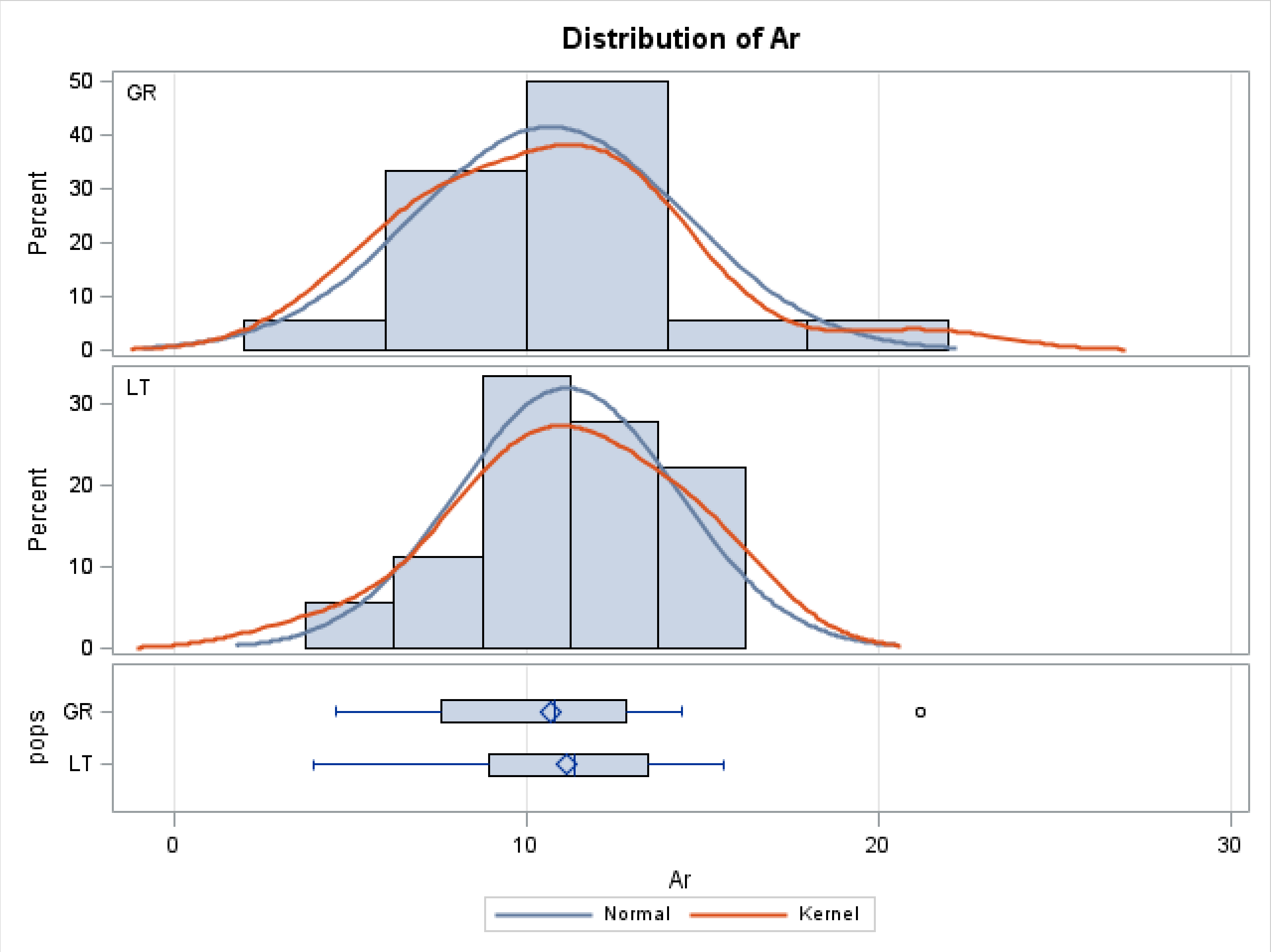
Distribution of Ar
GR
LT
Percent
pops
Ar
Normal
Kernel

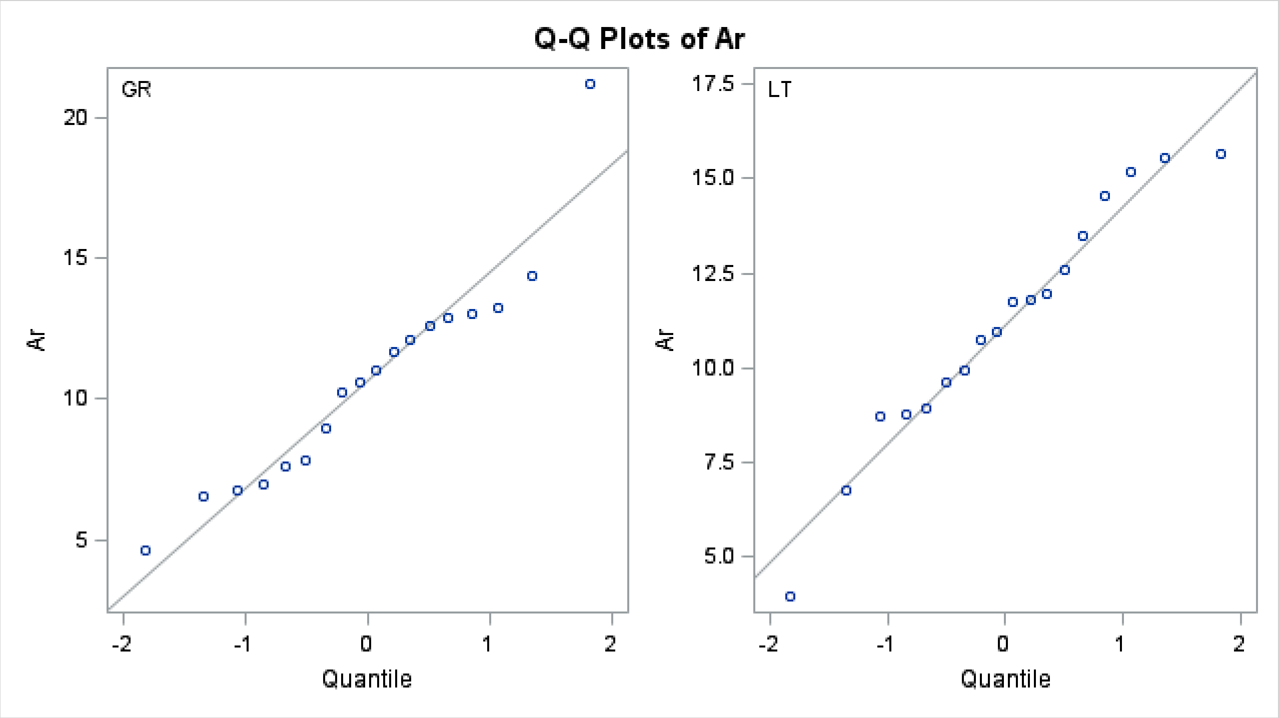
Q-Q Plots of Ar
GR
LT
Ar
Quantile

Table S7. Variable: rare alleles

pops	N	Mean	Std Dev	Std Err	Minimum	Maximum
**GR**	18	6.3333	3.4979	0.8245	2.0000	16.0000
**LT**	18	6.0556	2.5546	0.6021	2.0000	10.0000
**Diff (1-2)**		0.2778	3.0628	1.0209		

pops	Method	Mean	95% CL Mean		Std Dev	95% CL Std Dev	
**GR**		6.3333	4.5939	8.0728	3.4979	2.6248	5.2439
**LT**		6.0556	4.7852	7.3259	2.5546	1.9170	3.8298
**Diff (1-2)**	**Pooled**	0.2778	-1.7970	2.3526	3.0628	2.4774	4.0129
**Diff (1-2)**	**Satterthwaite**	0.2778	-1.8041	2.3597			

Method	Variances	DF	t Value	Pr > |t|
**Pooled**	Equal	34	0.27	0.7872
**Satterthwaite**	Unequal	31.118	0.27	0.7874

Equality of Variances

Method	Num DF	Den DF	F Value	Pr > F
**Folded F**	17	17	1.87	0.2053

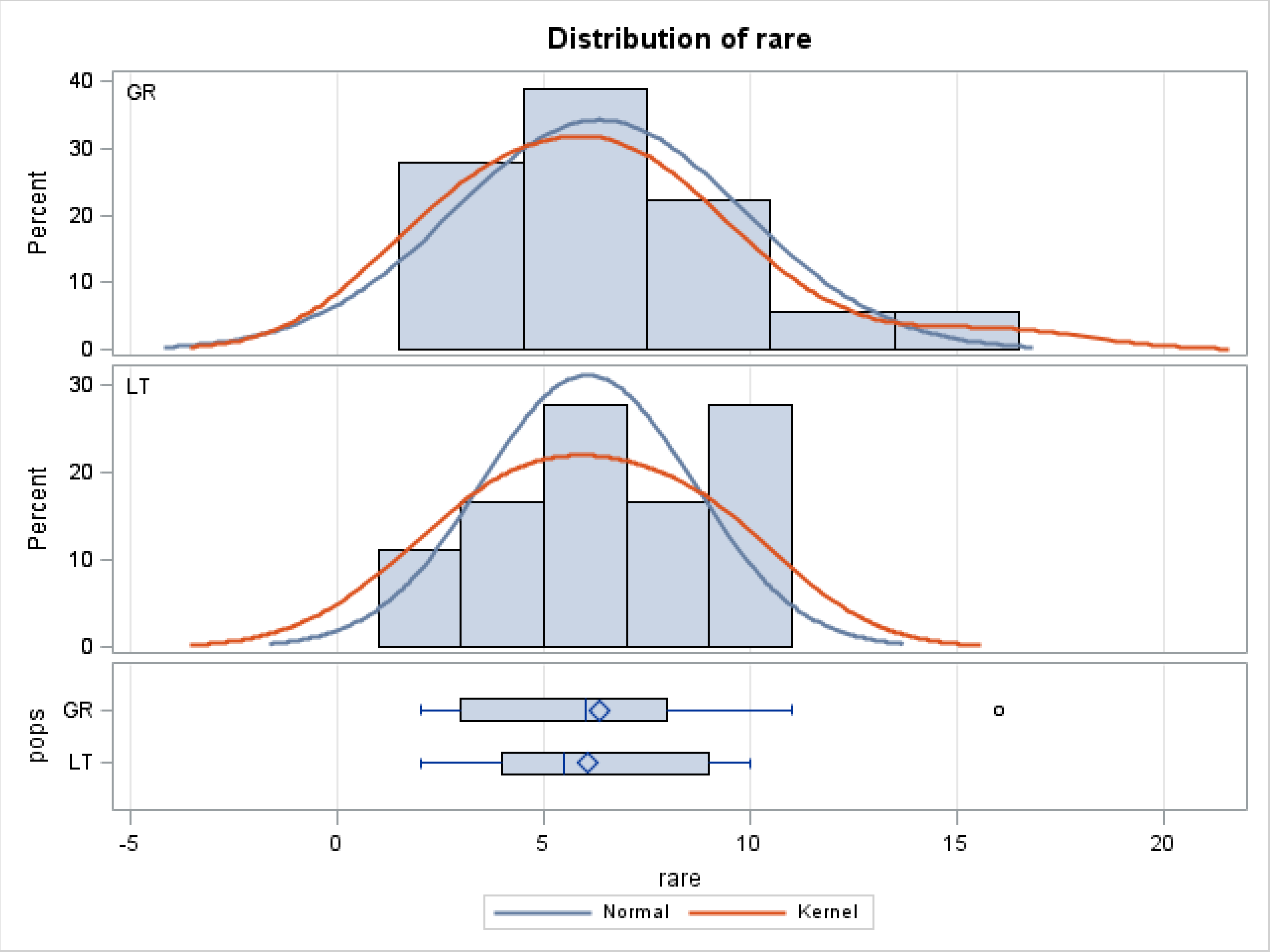
Distribution of rare
GR
LT
Percent
Percent
pops
rare
Normal
Kernel

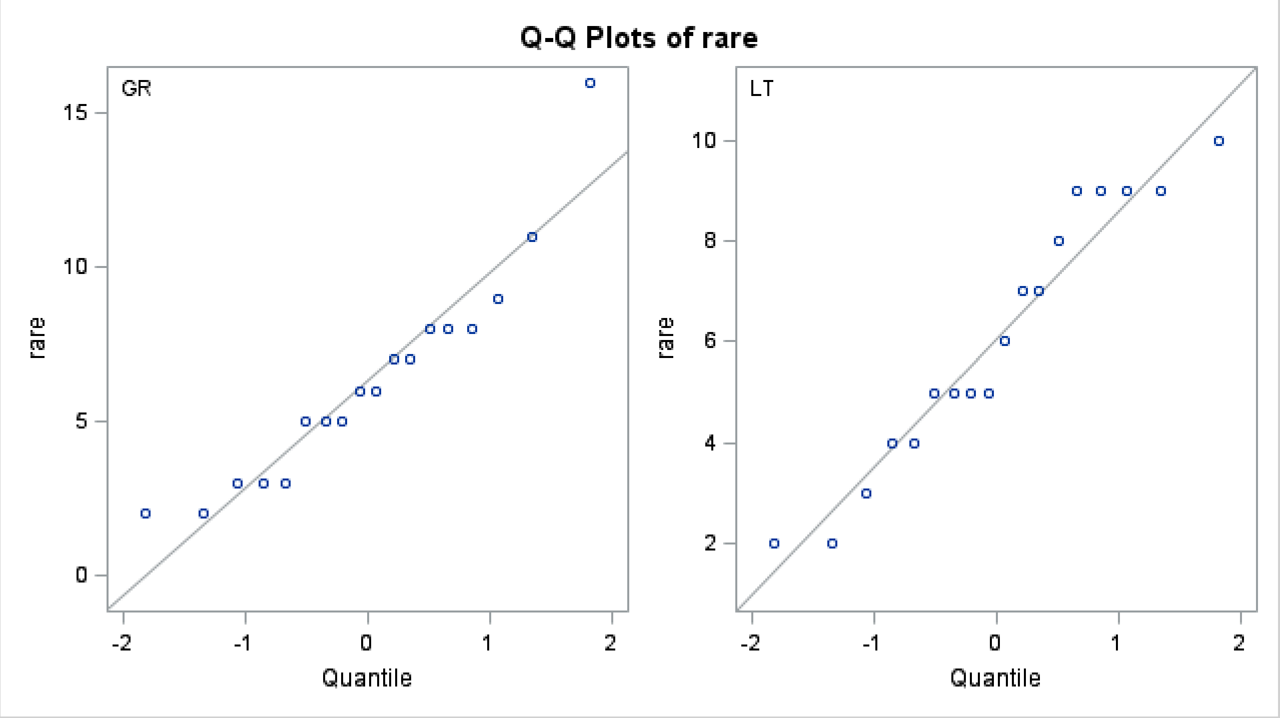
Q-Q Plots of rare
GR
LT
rare
rare
Quantile
Quantile

Table S8. Variable: private alleles

pops	N	Mean	Std Dev	Std Err	Minimum	Maximum
GR	18	2.0556	2.0996	0.4949	0	8.0000
LT	18	2.5000	1.3394	0.3157	0	5.0000
Diff (1-2)		-0.4444	1.7611	0.5870		

pops	Method	Mean	95% CL Mean		Std Dev	95% CL Std Dev	
GR		2.0556	1.0114	3.0997	2.0996	1.5755	3.1477
LT		2.5000	1.8339	3.1661	1.3394	1.0051	2.0080
Diff (1-2)	Pooled	-0.4444	-1.6374	0.7485	1.7611	1.4245	2.3073
Diff (1-2)	Satterthwaite	-0.4444	-1.6453	0.7564			

Method	Variances	DF	t Value	Pr > |t|
Pooled	Equal	34	-0.76	0.4542
Satterthwaite	Unequal	28.871	-0.76	0.4551

Equality of Variances

Method	Num DF	Den DF	F Value	Pr > F
Folded F	17	17	2.46	0.0723

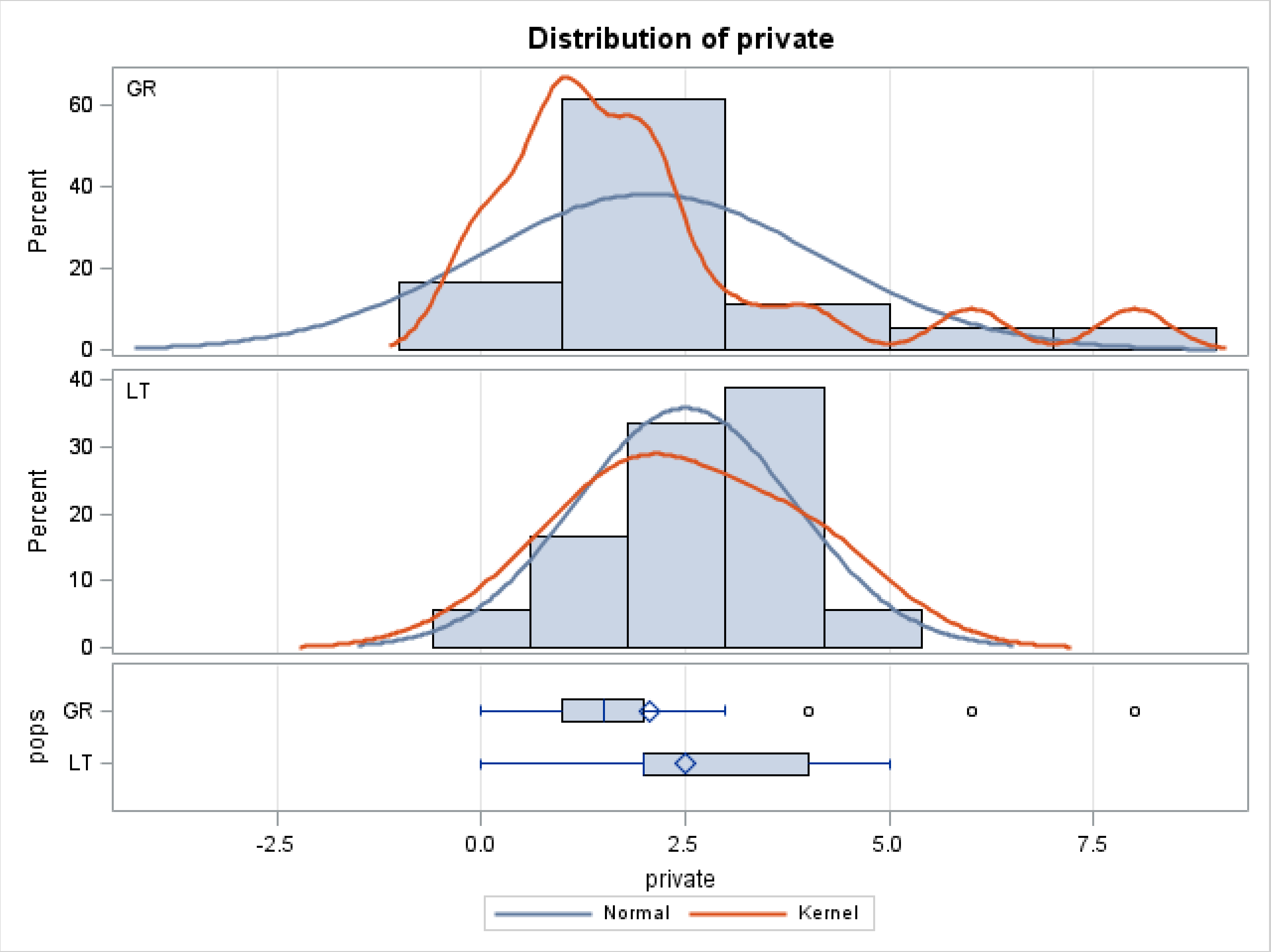
Distribution of private
GR
LT
Percent
pops
private
Normal
Kernel

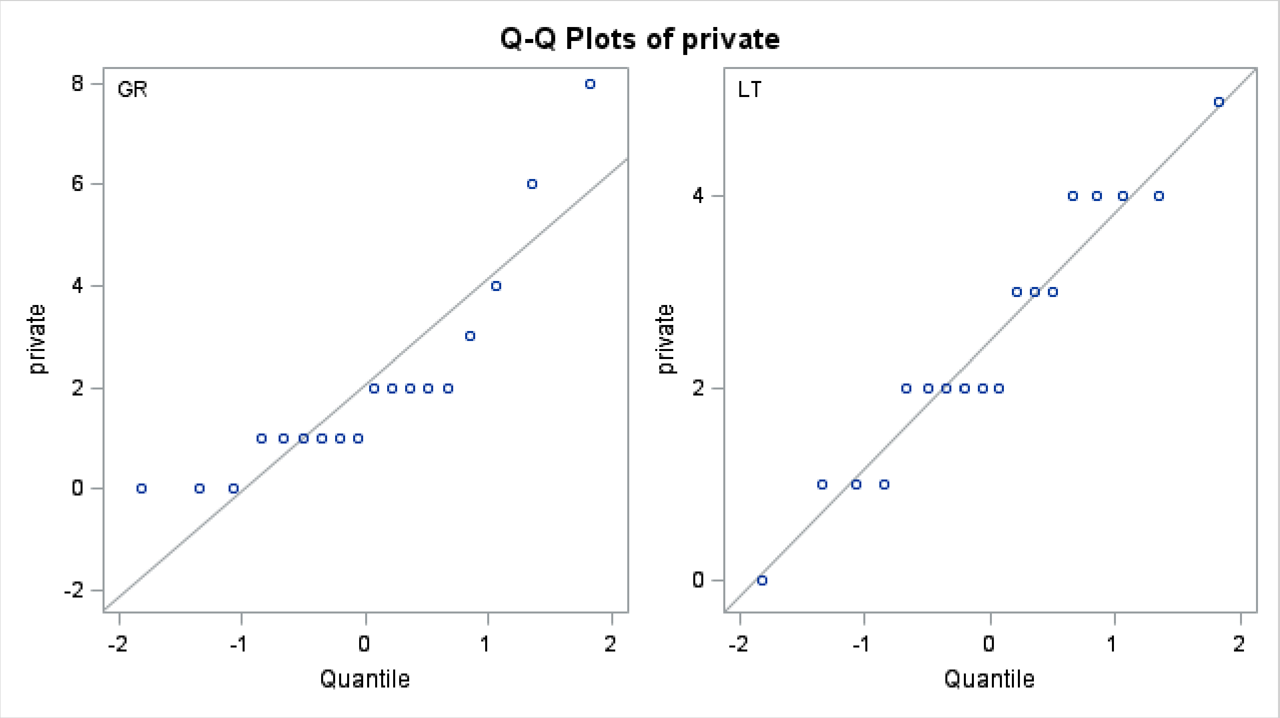
Q-Q Plots of private
GR
LT
private
Quantile

Picea abies

The SAS System

The TTEST Procedure

Table S9. Variable: Na

pops	N	Mean	Std Dev	Std Err	Minimum	Maximum
**GR**	18	18.3333	9.3494	2.2037	6.0000	34.0000
**LT**	18	16.4444	6.9216	1.6314	6.0000	28.0000
**Diff (1-2)**		1.8889	8.2256	2.7419		

pops	Method	Mean	95% CL Mean		Std Dev	95% CL Std Dev	
**GR**		18.3333	13.6840	22.9827	9.3494	7.0157	14.0161
**LT**		16.4444	13.0024	19.8865	6.9216	5.1939	10.3765
**Diff (1-2)**	**Pooled**	1.8889	-3.6832	7.4610	8.2256	6.6534	10.7772
**Diff (1-2)**	**Satterthwaite**	1.8889	-3.7008	7.4786			

Method	Variances	DF	t Value	Pr > |t|
**Pooled**	Equal	34	0.69	0.4956
**Satterthwaite**	Unequal	31.33	0.69	0.4960

Equality of Variances

Method	Num DF	Den DF	F Value	Pr > F
**Folded F**	17	17	1.82	0.2252

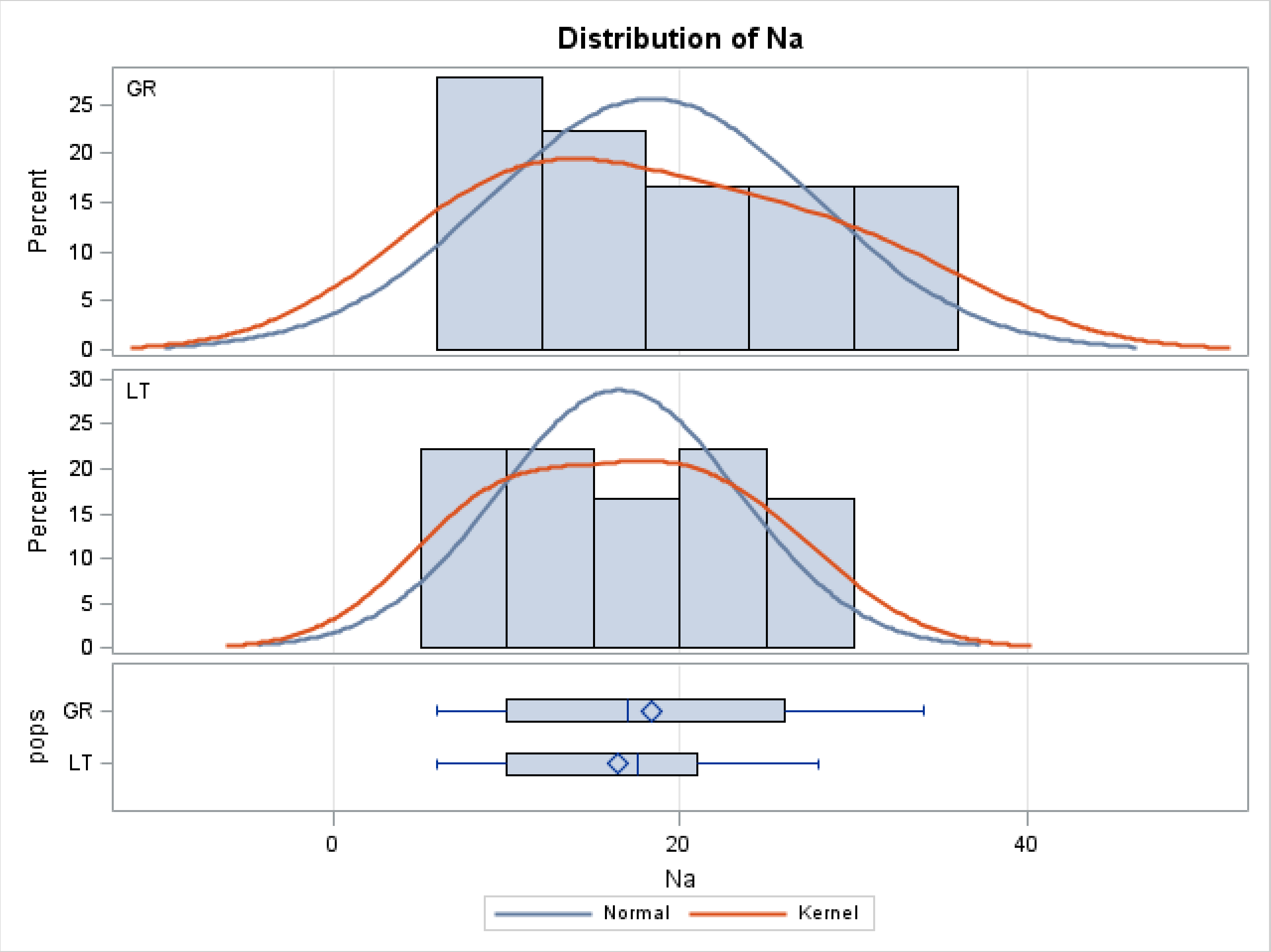

Distribution of Na
GR
LT
Percent
pops
Na
Normal
Kernel

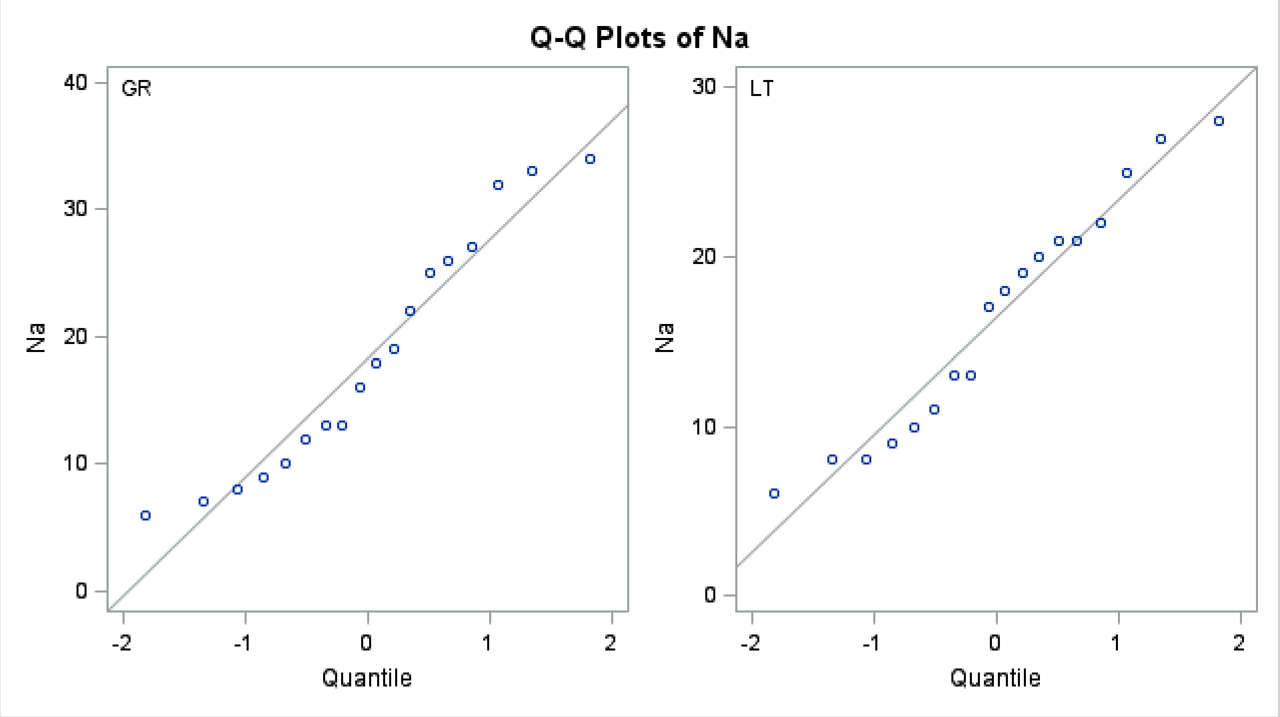

Q-Q Plots of Na
GR
LT
Na
Quantile

Table S10. Variable: Ne

pops	N	Mean	Std Dev	Std Err	Minimum	Maximum
**GR**	18	7.8114	6.0560	1.4274	1.6627	20.7879
**LT**	18	5.5946	3.5291	0.8318	1.5107	15.5152
**Diff (1-2)**		2.2168	4.9563	1.6521		

pops	Method	Mean	95% CL Mean		Std Dev	95% CL Std Dev	
**GR**		7.8114	4.7998	10.8230	6.0560	4.5443	9.0788
**LT**		5.5946	3.8396	7.3496	3.5291	2.6482	5.2907
**Diff (1-2)**	**Pooled**	2.2168	-1.1406	5.5743	4.9563	4.0090	6.4938
**Diff (1-2)**	**Satterthwaite**	2.2168	-1.1710	5.6046			

Method	Variances	DF	t Value	Pr > |t|
**Pooled**	Equal	34	1.34	0.1885
**Satterthwaite**	Unequal	27.353	1.34	0.1907

Equality of Variances

Method	Num DF	Den DF	F Value	Pr > F
**Folded F**	17	17	2.94	0.0320

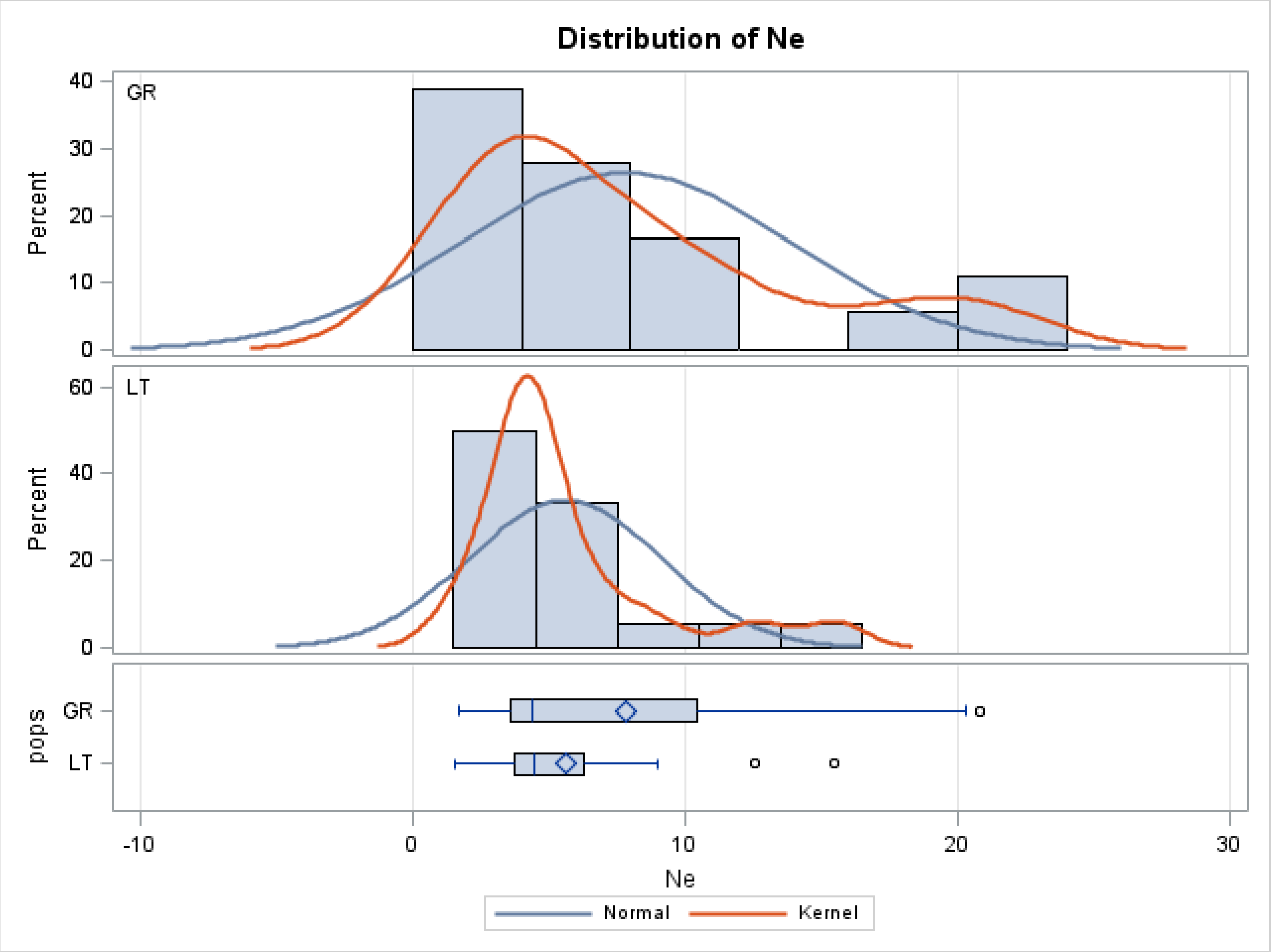
Distribution of Ne
GR
LT
Percent
pops
Ne
Normal
Kernel
-10
0
10
20
30
40
60

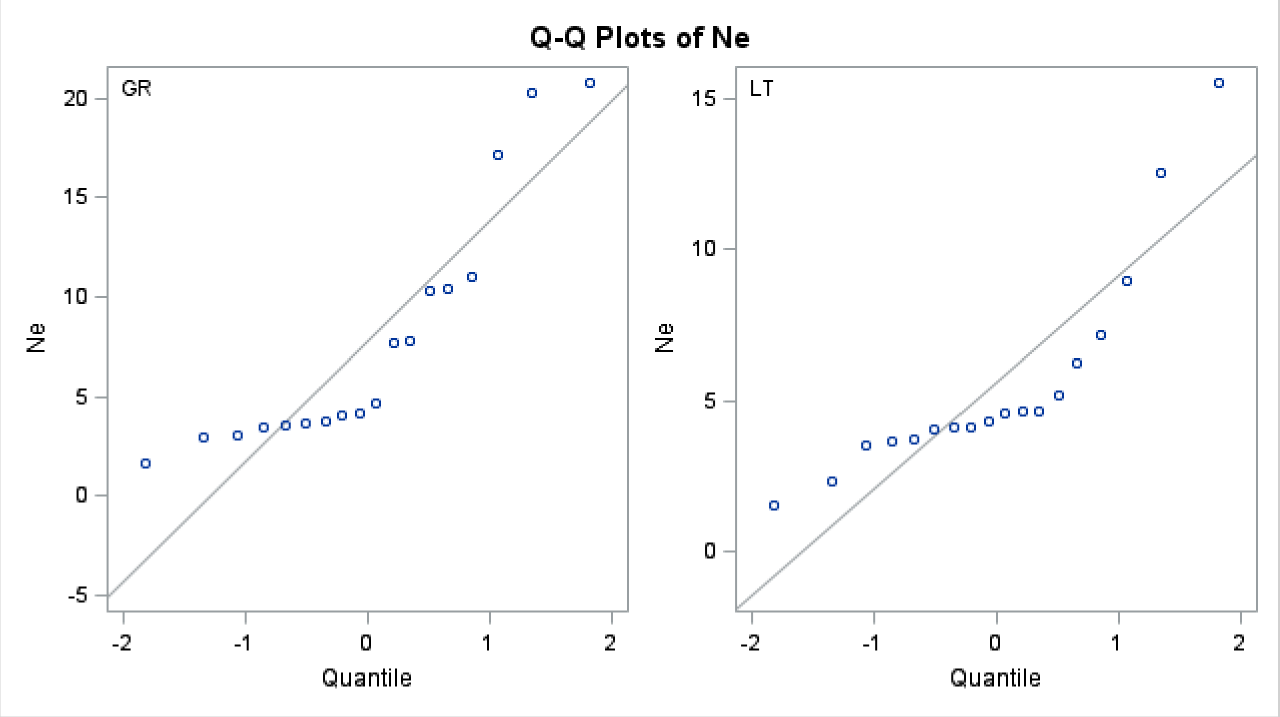
Q-Q Plots of Ne
GR
LT
Ne
Quantile
-2
-1
0
1
2
-5
5
10
15
20

Table S11. Variable: Ho

pops	N	Mean	Std Dev	Std Err	Minimum	Maximum
**GR**	18	0.8418	0.1690	0.0398	0.4490	1.0000
**LT**	18	0.8257	0.1849	0.0436	0.3375	1.0000
**Diff (1-2)**		0.0161	0.1771	0.0590		

pops	Method	Mean	95% CL Mean		Std Dev	95% CL Std Dev	
**GR**		0.8418	0.7578	0.9259	0.1690	0.1268	0.2534
**LT**		0.8257	0.7338	0.9176	0.1849	0.1387	0.2772
**Diff (1-2)**	**Pooled**	0.0161	-0.1039	0.1361	0.1771	0.1433	0.2321
**Diff (1-2)**	**Satterthwaite**	0.0161	-0.1039	0.1361			

Method	Variances	DF	t Value	Pr > |t|
**Pooled**	Equal	34	0.27	0.7865
**Satterthwaite**	Unequal	33.73	0.27	0.7865

Equality of Variances

Method	Num DF	Den DF	F Value	Pr > F
**Folded F**	17	17	1.20	0.7159

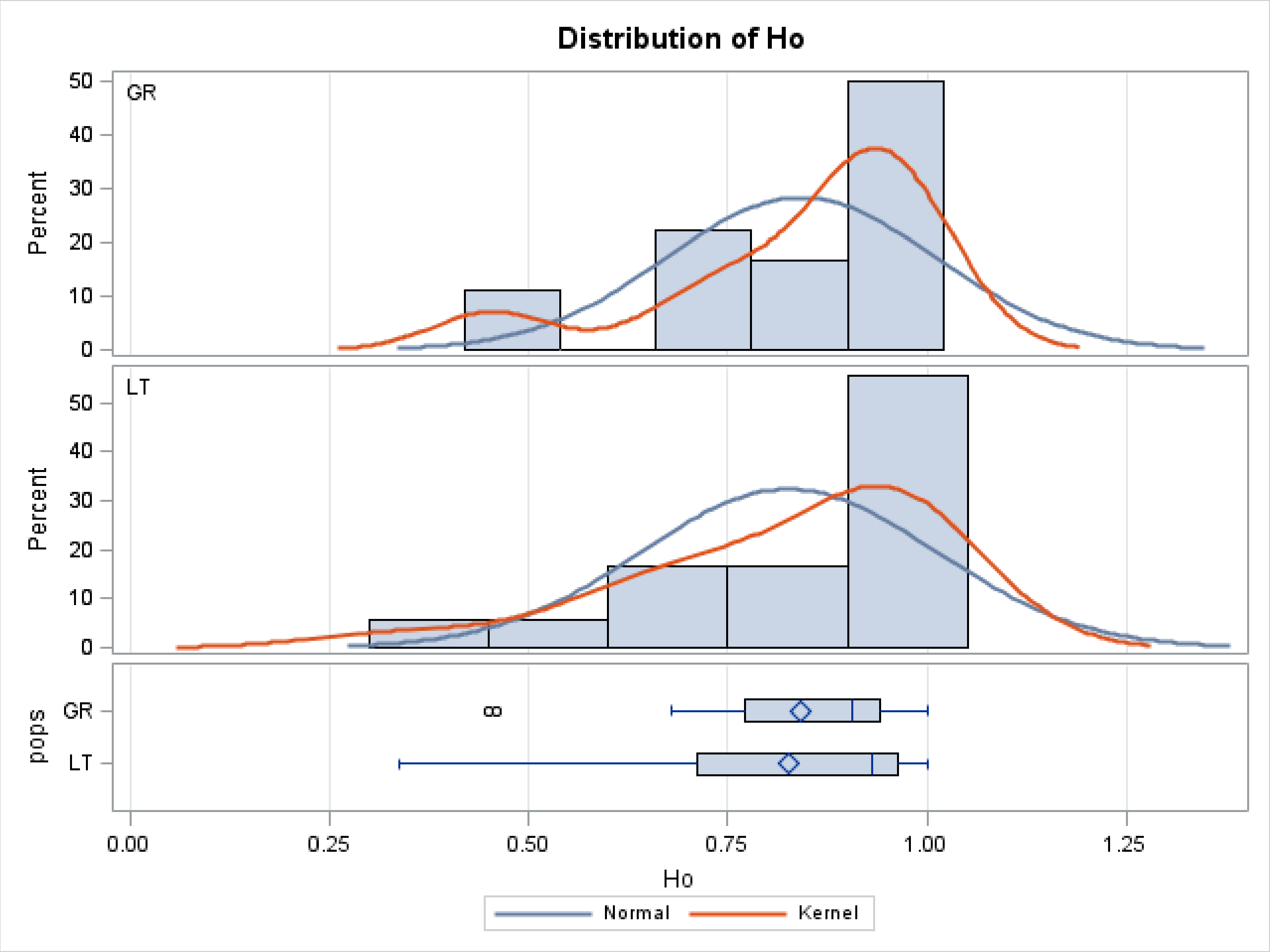

Distribution of Ho
GR
LT
Percent
pops
Ho
Normal
Kernel

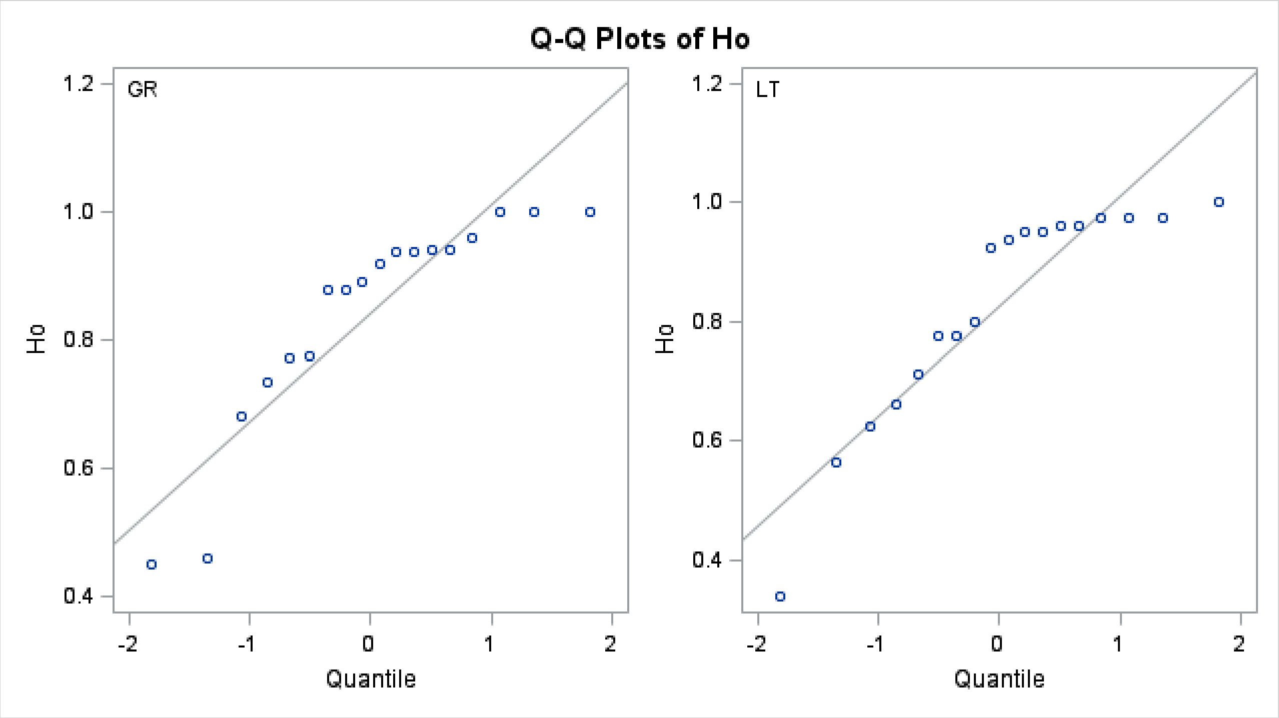

Q-Q Plots of Ho
GR
LT
Ho
Quantile

Table S12. Variable: He

pops	N	Mean	Std Dev	Std Err	Minimum	Maximum
**GR**	18	0.7911	0.1394	0.0329	0.3986	0.9519
**LT**	18	0.7617	0.1357	0.0320	0.3380	0.9355
**Diff (1-2)**		0.0295	0.1375	0.0458		

pops	Method	Mean	95% CL Mean		Std Dev	95% CL Std Dev	
**GR**		0.7911	0.7218	0.8604	0.1394	0.1046	0.2090
**LT**		0.7617	0.6942	0.8291	0.1357	0.1018	0.2034
**Diff (1-2)**	**Pooled**	0.0295	-0.0637	0.1226	0.1375	0.1112	0.1802
**Diff (1-2)**	**Satterthwaite**	0.0295	-0.0637	0.1226			

Method	Variances	DF	t Value	Pr > |t|
**Pooled**	Equal	34	0.64	0.5249
**Satterthwaite**	Unequal	33.975	0.64	0.5249

Equality of Variances

Method	Num DF	Den DF	F Value	Pr > F
**Folded F**	17	17	1.06	0.9125

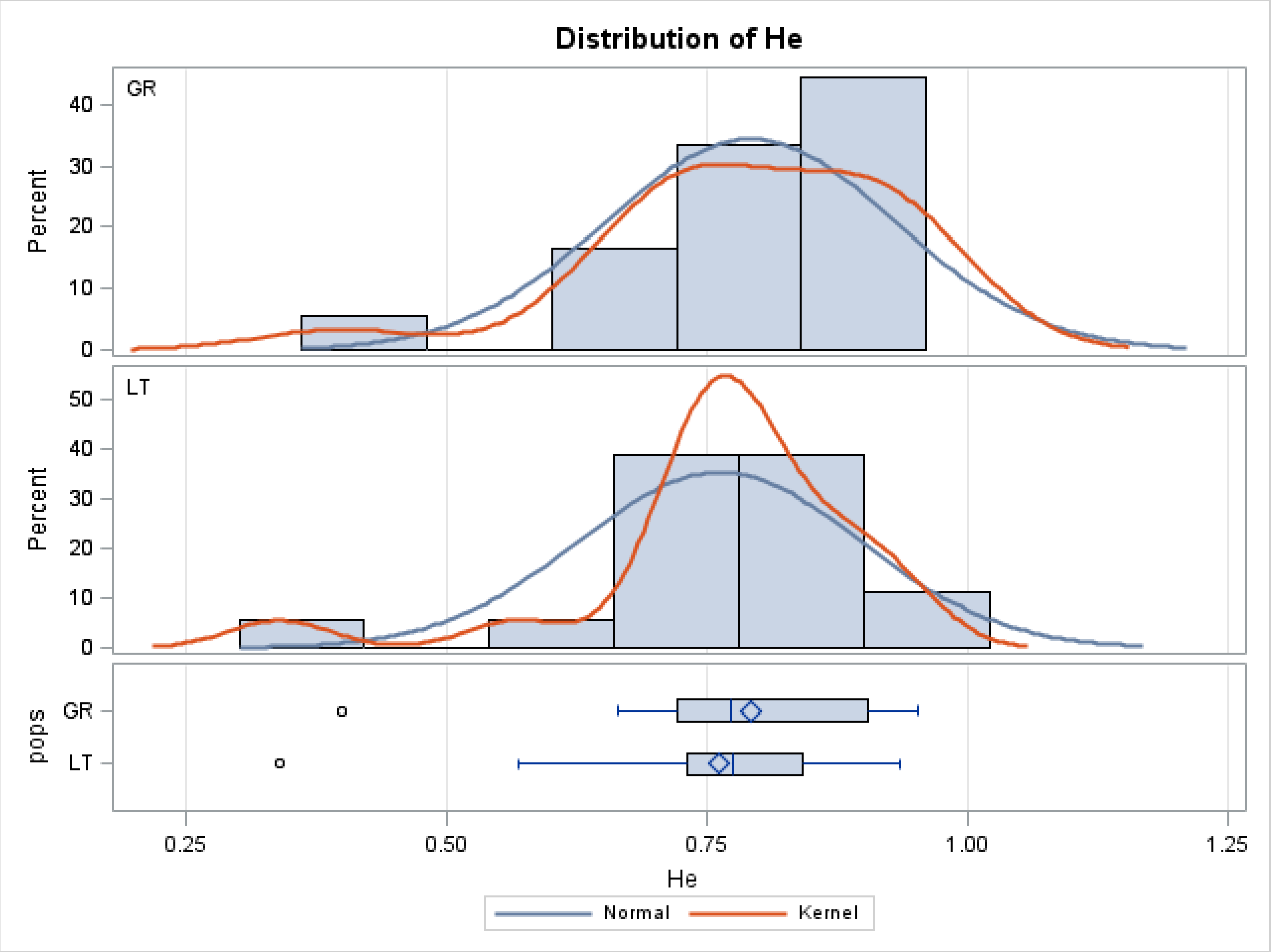

Distribution of He
GR
LT
Percent
pops
He
Normal
Kernel
0.25
0.50
0.75
1.00
1.25

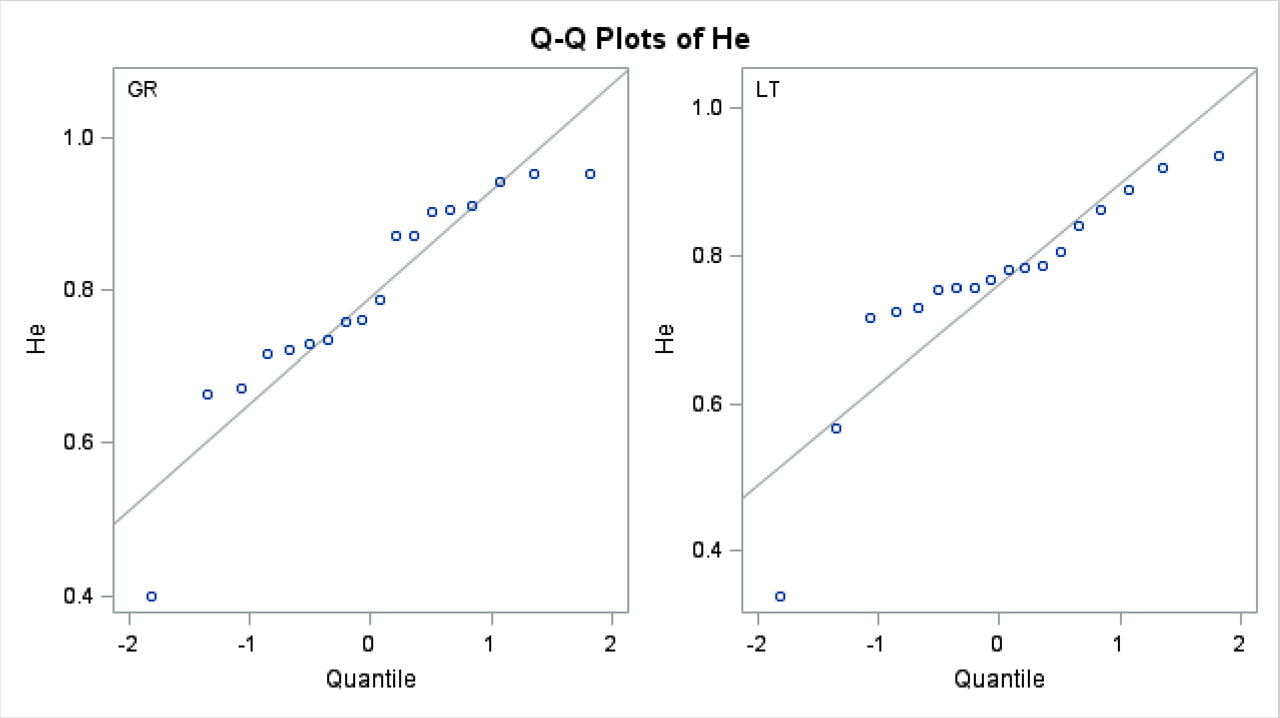

Q-Q Plots of He
GR
LT
He
Quantile

Table S13. Variable: Fis

pops	N	Mean	Std Dev	Std Err	Minimum	Maximum
**GR**	18	-0.0705	0.2224	0.0524	-0.4060	0.4030
**LT**	18	-0.0897	0.2288	0.0539	-0.6710	0.4040
**Diff (1-2)**		0.0192	0.2256	0.0752		

pops	Method	Mean	95% CL Mean		Std Dev	95% CL Std Dev	
**GR**		-0.0705	-0.1811	0.0401	0.2224	0.1669	0.3334
**LT**		-0.0897	-0.2035	0.0240	0.2288	0.1717	0.3429
**Diff (1-2)**	**Pooled**	0.0192	-0.1336	0.1720	0.2256	0.1825	0.2956
**Diff (1-2)**	**Satterthwaite**	0.0192	-0.1336	0.1720			

Method	Variances	DF	t Value	Pr > |t|
**Pooled**	Equal	34	0.26	0.7998
**Satterthwaite**	Unequal	33.973	0.26	0.7998

Equality of Variances

Method	Num DF	Den DF	F Value	Pr > F
**Folded F**	17	17	1.06	0.9083

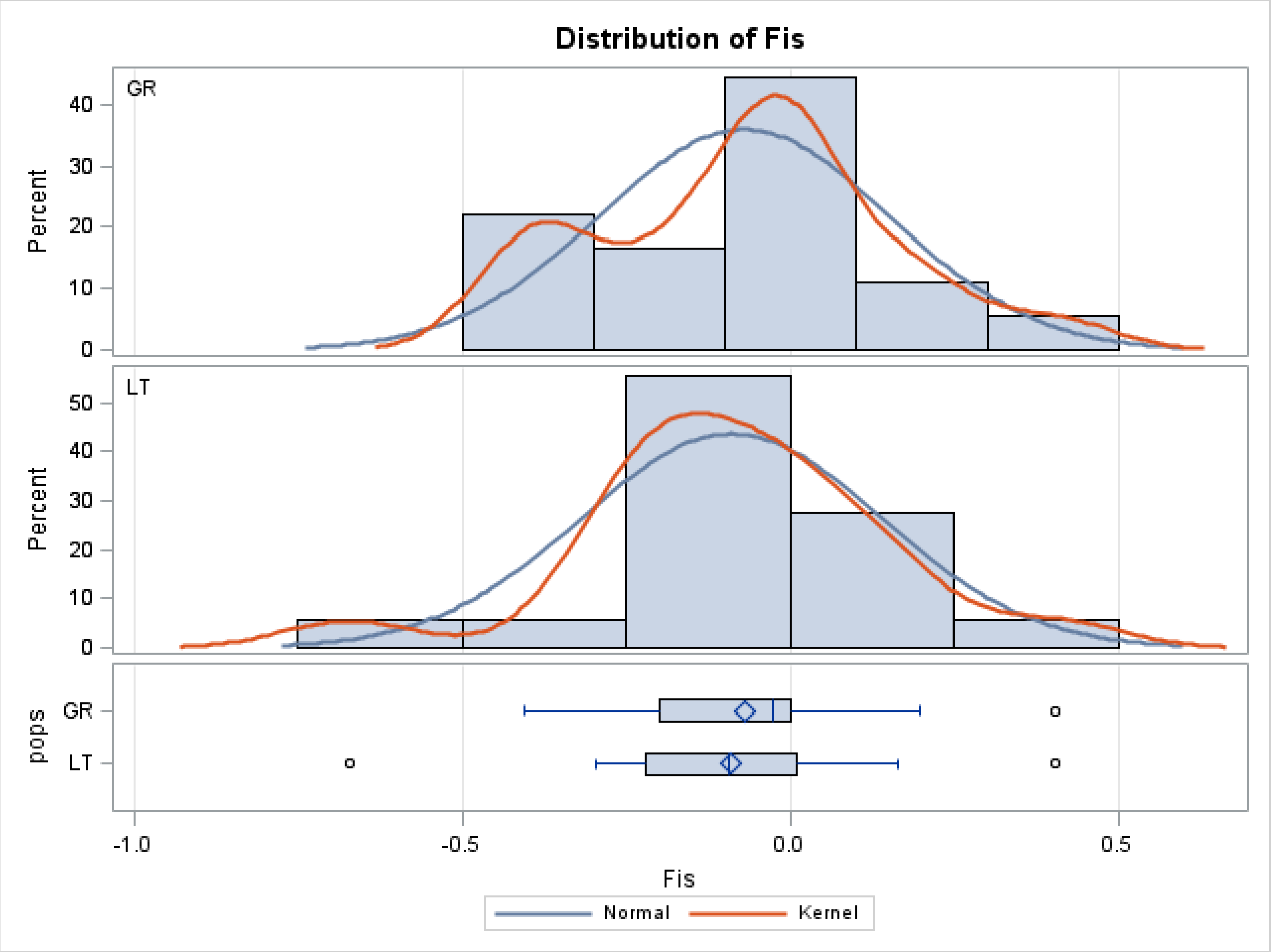

Distribution of Fis
GR
LT
Percent
pops
-1.0
-0.5
0.0
0.5
Fis
Normal
Kernel

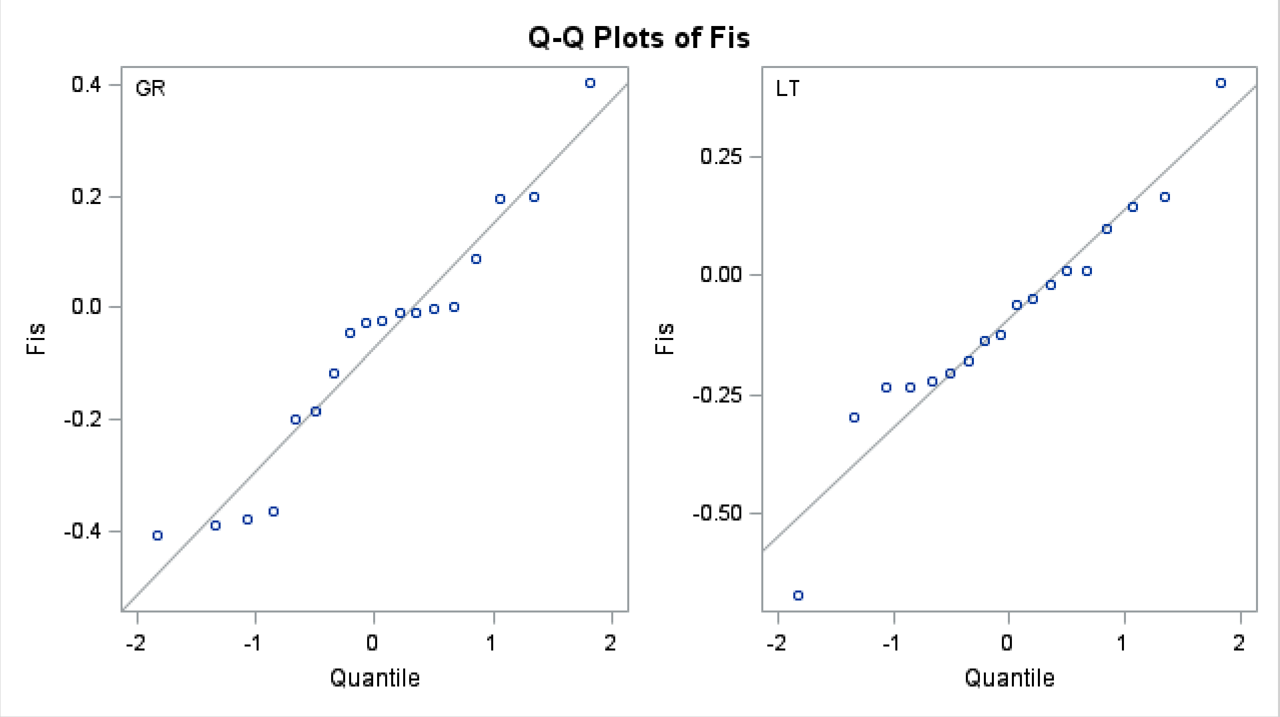

Q-Q Plots of Fis
GR
LT
Fis
Quantile

Table S14. Variable: Ar

pops	N	Mean	Std Dev	Std Err	Minimum	Maximum
**GR**	18	17.6112	8.9722	2.1148	6.0000	32.6120
**LT**	18	13.7051	5.6764	1.3379	5.5090	24.5930
**Diff (1-2)**		3.9061	7.5074	2.5025		

pops	Method	Mean	95% CL Mean		Std Dev	95% CL Std Dev	
**GR**		17.6112	13.1494	22.0730	8.9722	6.7326	13.4507
**LT**		13.7051	10.8823	16.5279	5.6764	4.2595	8.5097
**Diff (1-2)**	**Pooled**	3.9061	-1.1795	8.9917	7.5074	6.0725	9.8362
**Diff (1-2)**	**Satterthwaite**	3.9061	-1.2141	9.0263			

Method	Variances	DF	t Value	Pr > |t|
**Pooled**	Equal	34	1.56	0.1278
**Satterthwaite**	Unequal	28.73	1.56	0.1295

Equality of Variances

Method	Num DF	Den DF	F Value	Pr > F
**Folded F**	17	17	2.50	0.0673

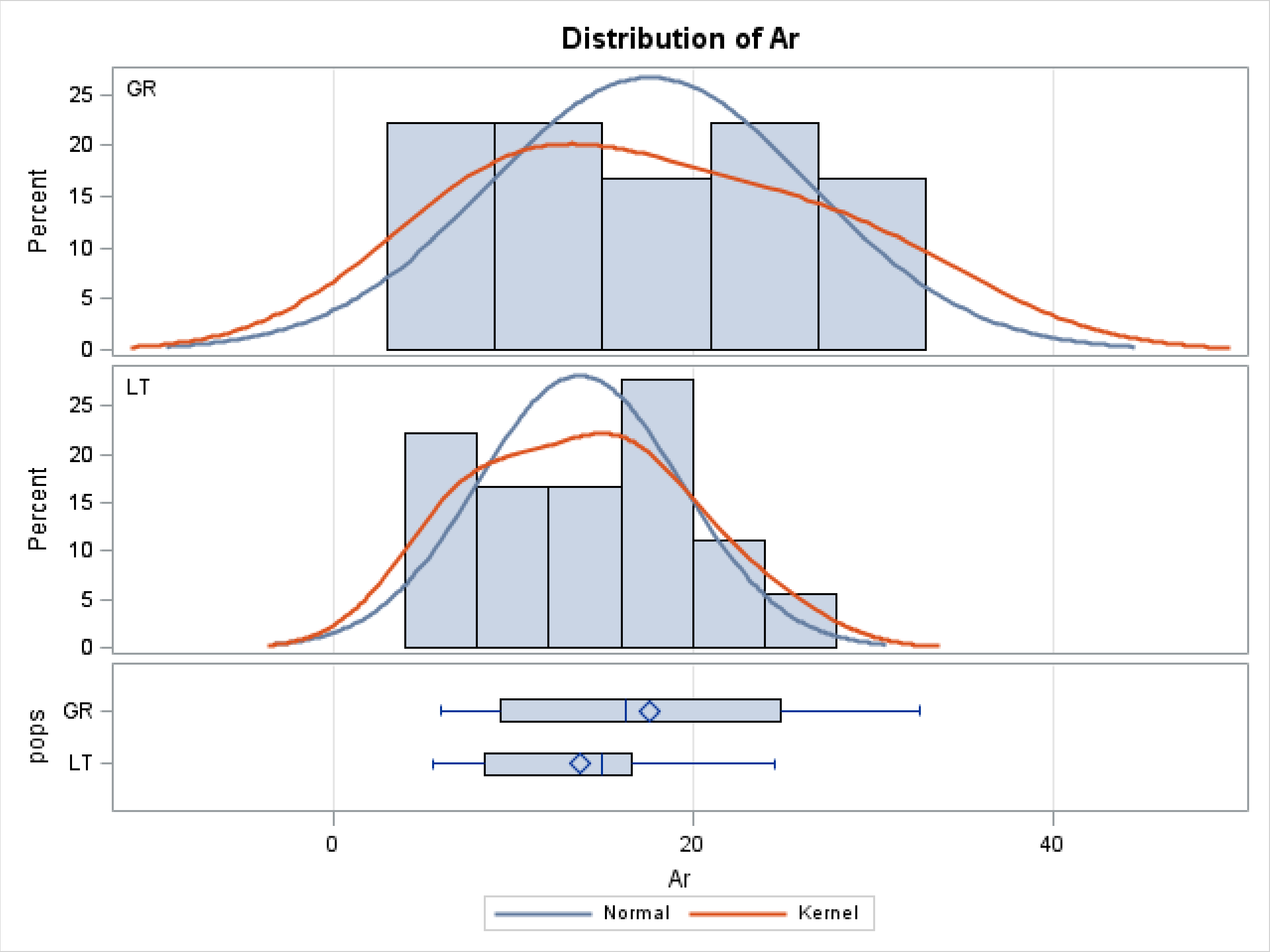
Distribution of Ar
GR
LT
Percent
25
20
15
10
5
0
pops
Ar
0
20
40
Normal
Kernel

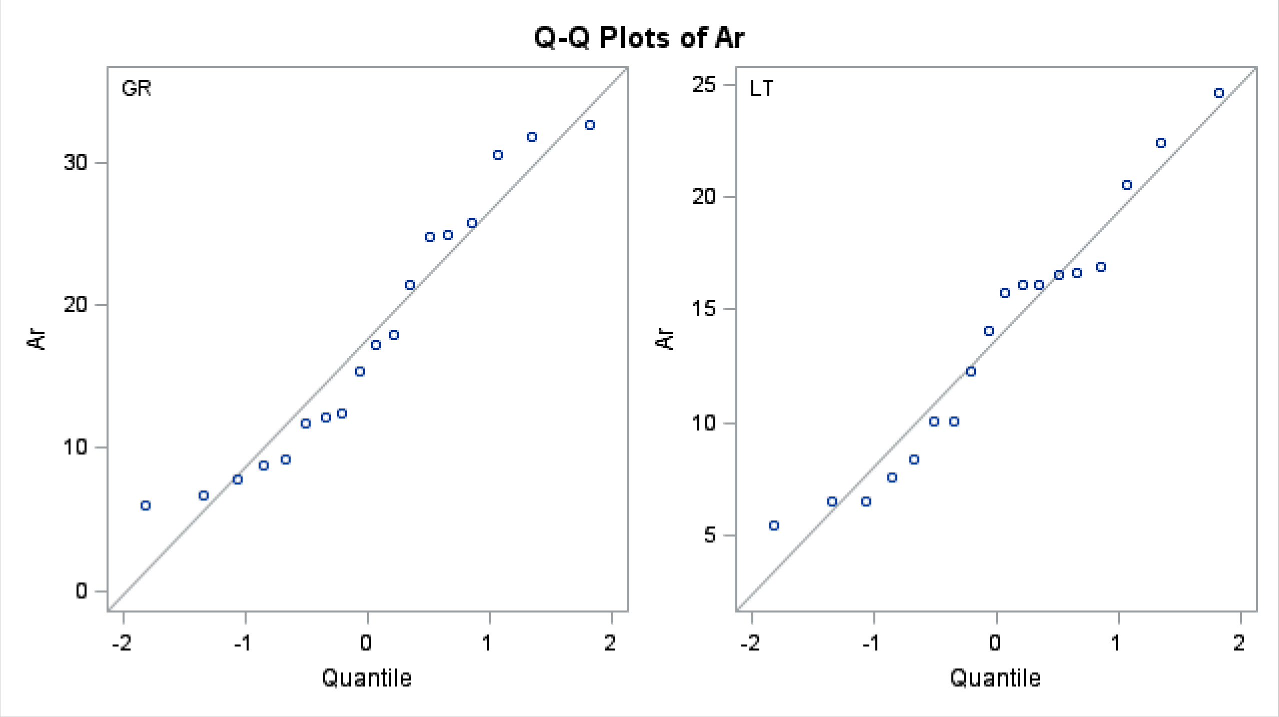
Q-Q Plots of Ar
GR
LT
Ar
Quantile
-2
-1
0
1
2
30
20
10
0
25
15
5

Table S15. Variable: rare alleles

pops	N	Mean	Std Dev	Std Err	Minimum	Maximum
GR	18	13.8333	8.5285	2.0102	1.0000	28.0000
LT	18	12.3889	6.1657	1.4533	3.0000	22.0000
Diff (1-2)		1.4444	7.4415	2.4805		

pops	Method	Mean	95% CL Mean		Std Dev	95% CL Std Dev	
GR		13.8333	9.5922	18.0745	8.5285	6.3997	12.7854
LT		12.3889	9.3227	15.4550	6.1657	4.6267	9.2433
Diff (1-2)	Pooled	1.4444	-3.5965	6.4854	7.4415	6.0192	9.7499
Diff (1-2)	Satterthwaite	1.4444	-3.6148	6.5037			

Method	Variances	DF	t Value	Pr > |t|
Pooled	Equal	34	0.58	0.5642
Satterthwaite	Unequal	30.958	0.58	0.5646

Equality of Variances

Method	Num DF	Den DF	F Value	Pr > F
Folded F	17	17	1.91	0.1912

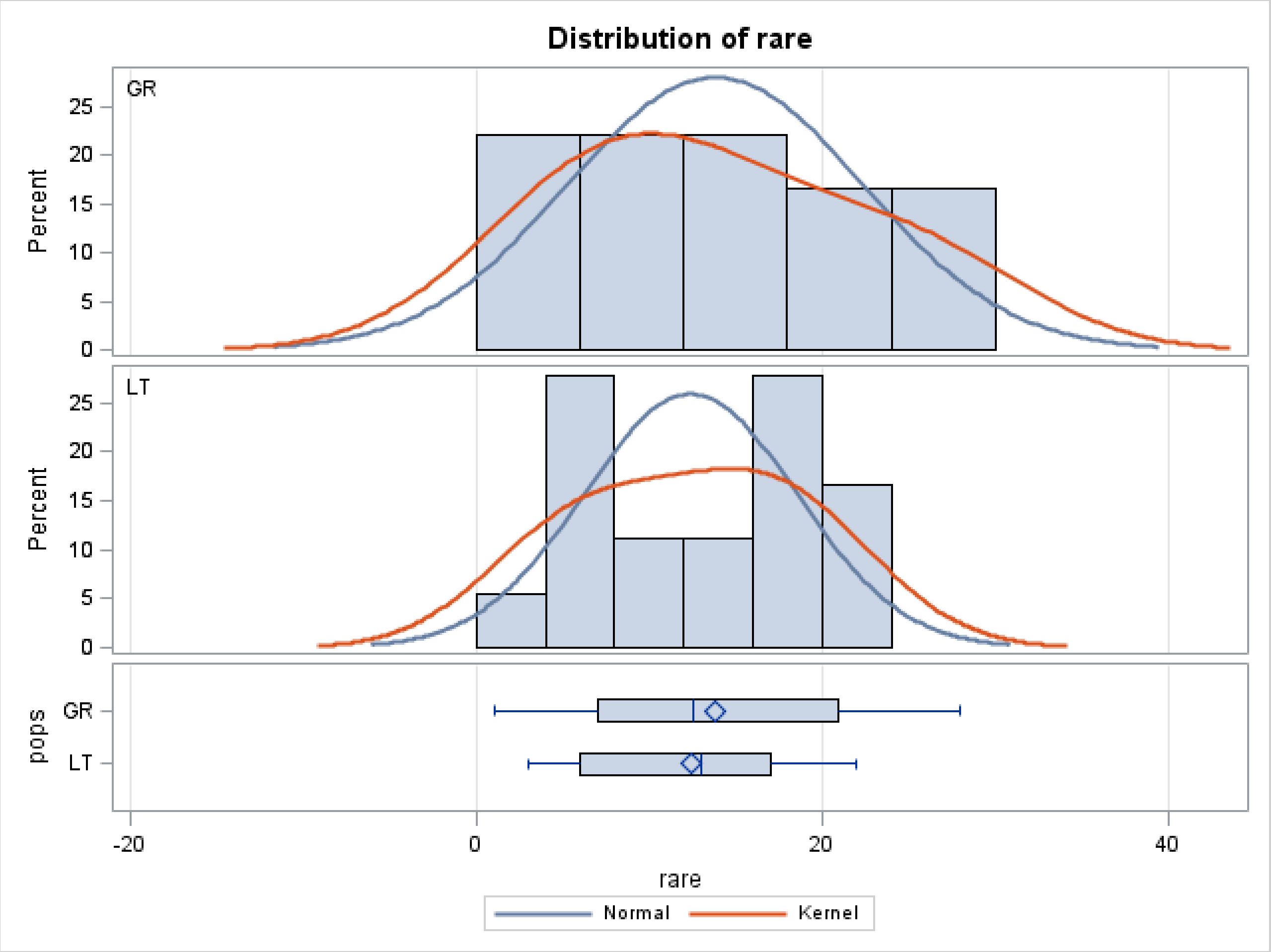
Distribution of rare
GR
LT
Percent
pops
rare
Normal
Kernel

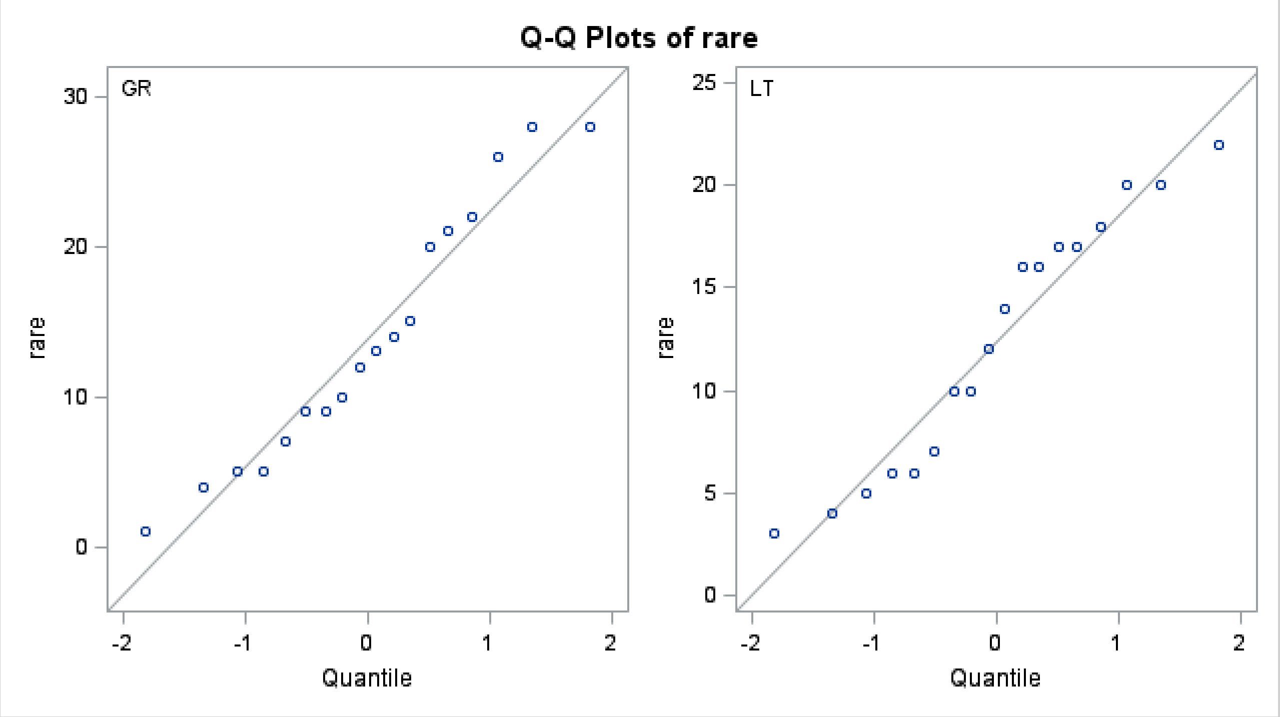
Q-Q Plots of rare
GR
LT
rare
Quantile

Table S16. Variable: private alleles

pops	N	Mean	Std Dev	Std Err	Minimum	Maximum
**GR**	18	6.5000	5.3934	1.2712	0	19.0000
**LT**	18	4.6111	3.3279	0.7844	1.0000	11.0000
**Diff (1-2)**		1.8889	4.4813	1.4938		

pops	Method	Mean	95% CL Mean		Std Dev	95% CL Std Dev	
**GR**		6.5000	3.8179	9.1821	5.3934	4.0471	8.0854
**LT**		4.6111	2.9562	6.2661	3.3279	2.4972	4.9891
**Diff (1-2)**	**Pooled**	1.8889	-1.1468	4.9246	4.4813	3.6248	5.8714
**Diff (1-2)**	**Satterthwaite**	1.8889	-1.1694	4.9472			

Method	Variances	DF	t Value	Pr > |t|
**Pooled**	Equal	34	1.26	0.2146
**Satterthwaite**	Unequal	28.306	1.26	0.2164

Equality of Variances

Method	Num DF	Den DF	F Value	Pr > F
**Folded F**	17	17	2.63	0.0541

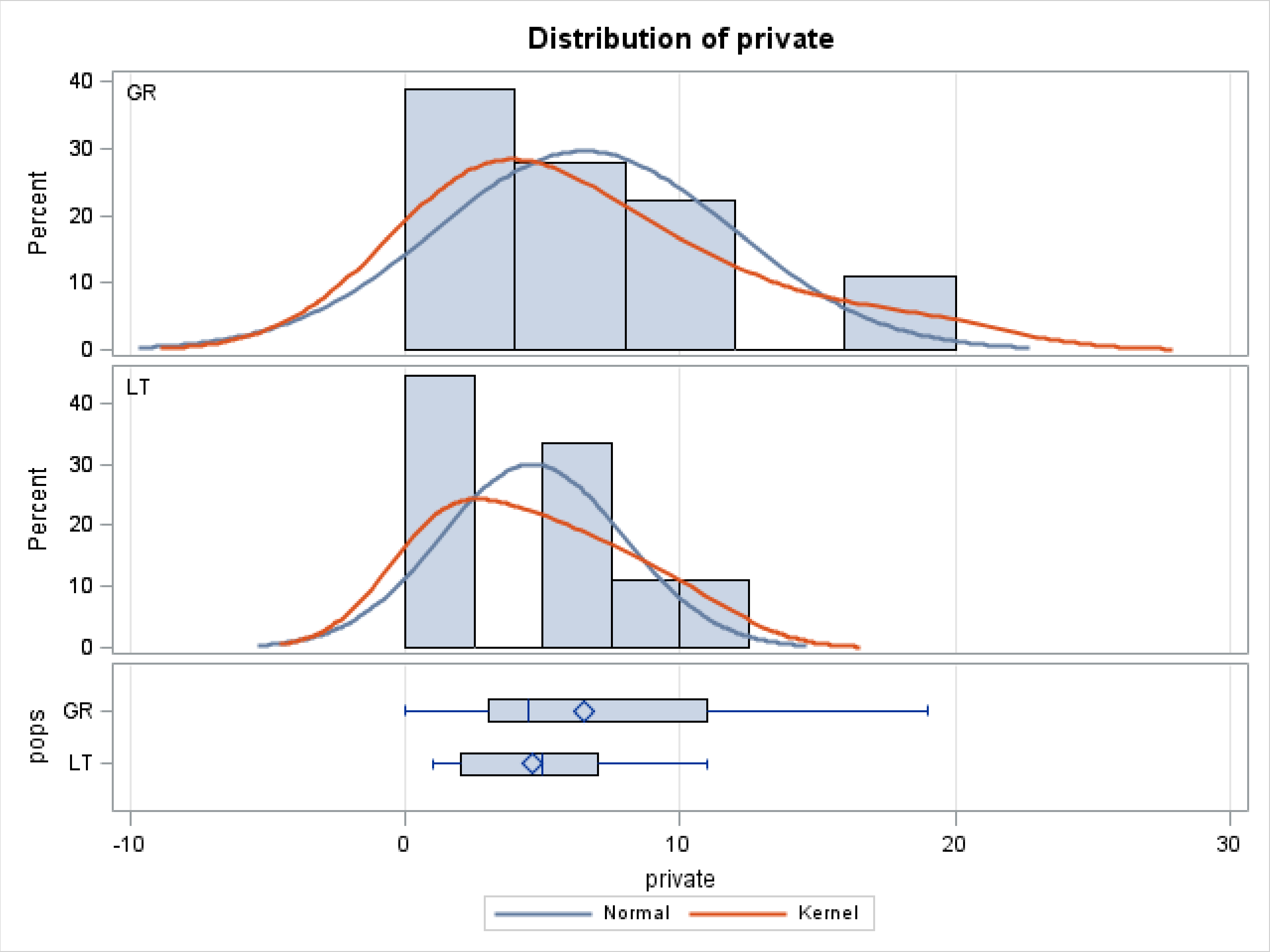

Distribution of private
GR
LT
Percent
pops
private
Normal
Kernel

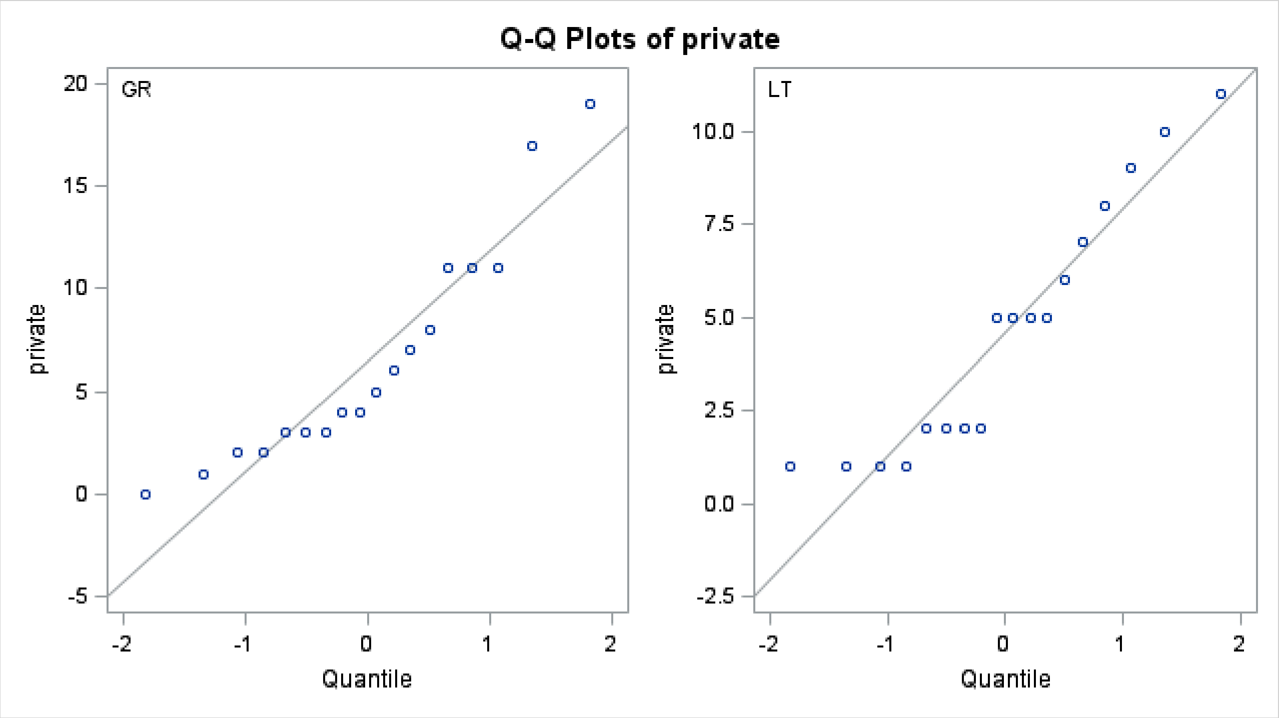

Q-Q Plots of private
GR
LT
private
Quantile
